# The Evolutionary Dynamics of Protein-Protein Interaction Networks Inferred from the Reconstruction of Ancient Networks

**DOI:** 10.1371/journal.pone.0058134

**Published:** 2013-03-20

**Authors:** Yuliang Jin, Dmitrij Turaev, Thomas Weinmaier, Thomas Rattei, Hernán A. Makse

**Affiliations:** 1 Levich Institute and Physics Department, City College of New York, New York, New York, United States of America; 2 Department of Computational Systems Biology, University of Vienna, Vienna, Austria; University of Maryland, United States of America

## Abstract

Cellular functions are based on the complex interplay of proteins, therefore the structure and dynamics of these protein-protein interaction (PPI) networks are the key to the functional understanding of cells. In the last years, large-scale PPI networks of several model organisms were investigated. A number of theoretical models have been developed to explain both the network formation and the current structure. Favored are models based on duplication and divergence of genes, as they most closely represent the biological foundation of network evolution. However, studies are often based on simulated instead of empirical data or they cover only single organisms. Methodological improvements now allow the analysis of PPI networks of multiple organisms simultaneously as well as the direct modeling of ancestral networks. This provides the opportunity to challenge existing assumptions on network evolution. We utilized present-day PPI networks from integrated datasets of seven model organisms and developed a theoretical and bioinformatic framework for studying the evolutionary dynamics of PPI networks. A novel filtering approach using percolation analysis was developed to remove low confidence interactions based on topological constraints. We then reconstructed the ancient PPI networks of different ancestors, for which the ancestral proteomes, as well as the ancestral interactions, were inferred. Ancestral proteins were reconstructed using orthologous groups on different evolutionary levels. A stochastic approach, using the duplication-divergence model, was developed for estimating the probabilities of ancient interactions from today's PPI networks. The growth rates for nodes, edges, sizes and modularities of the networks indicate multiplicative growth and are consistent with the results from independent static analysis. Our results support the duplication-divergence model of evolution and indicate fractality and multiplicative growth as general properties of the PPI network structure and dynamics.

## Introduction

A living cell relies on a wide network of protein-protein interactions (PPIs) of structural and functional relevance, therefore the understanding of cell function is intrinsically tied to the understanding of this network. Technical advances in molecular and cellular biology and bioinformatics enabled extensive studies on protein-protein interaction networks (PIN) during the last decade. While a significant amount of data was collected during this time, theoretical analyses were focused on PINs from very few model organisms. Little is known about the comparability of results from different organisms as well as their transferability [1], [2]. General theoretical models explaining the formation, function and emerging properties of biological networks however often lack the connection to empirical data, making it difficult to validate the models [3]. Here we improve network theory for studying the evolutionary dynamics of PIN in multiple organisms.

### Experimental determination of protein-protein interaction networks

Multiple experimental methods for measuring PPI networks have been developed, like the yeast two-hybrid screen (Y2H) [4]–[6], the tandem affinity purification/mass spectrometry (TAP-MS) [7]–[9] and the protein-fragment complementation assay [10]. Each method has specific characteristics and limitations and therefore can provide only an incomplete view of the biological reality. For example, while TAP-MS detects stable complexes, weak and transient interactions are more readily detected by Y2H [11]. The precise determination of the error rates is difficult. For example, for Y2H experiments, estimates range from 10% to over 50% for the false positive rate and from 30% to 90% for the false negative rate [12], [13]. Furthermore, a bias is introduced by variations in the details of the Y2H protocol, such as the vectors used and the nature of the re-constituted transcription factor [14], [15]. For these reasons, the overlap between different studies is often small [6], [11], [12]. Possible approaches that can be applied for the selection of reliable interactions are reproducability, promiscuity, indirect support, conservation and topology [6], [16], whereas the best suited approach depends on the specific dataset.

Due to the volume of work and the methodological difficulties, genome-wide interactome studies were so far performed for only a limited number of organisms, among others *S. cerevisiae* [11], *H. sapiens* [17] and *A. thaliana* [18]. The results of these large-scale experiments and many other studies are collected in a number of databases like Mint [19], DIP [20], BioGrid [21] and IntAct [22]. These resources are partially redundant and use different database schemes, scores and identifiers. Integrating data from these sources for comprehensive analysis is therefore non-trivial. This problem is tackled e.g. by the STRING database, which incorporates different evidence sources for both physical and functional PPIs [23].

### Structure and topology of protein-protein interaction networks

For the characterization of the network structure, measures from network theory, like node degree, clustering coefficient or shortest path are used [24]. Based on these measures, observed networks can be assigned to different topological categories like random[25], small-world[26], hierarchical[27], fractal[28], and scale-free [24], [29].

PPI networks often show the small-world property, namely a short path length between any two nodes. The additional shortcuts in small-world networks affect the modularity, as well as the path length between proteins, and might for example influence signal transduction [26]. For small-world networks the scaling of the number of nodes and the average distance is exponential. It has also been shown that many complex networks show a scale-free topology, with the degree distribution following a power-law with the degree exponent 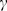 [30], [31]. A scale-free topology results in a high robustness of the network against perturbations [29].

PPI networks have also been shown to exhibit a highly modular structure, that is they contain substructures which are highly interconnected but have only few connections to nodes outside the module[24], [32]. The modular organization represents the higher-order correlations of the network structure beyond average properties, and has attracted great attention because it is closely related to the network functionality and robustness. For example, it has been shown that the modularity increases the overall robustness of the network by limiting the effect of local perturbations [24], [33], [34]. Along with the modular organization, the fractal and self-similar feature is empirically observed in many biological networks, such as the protein PPI networks[28], the biochemical reactions in metabolism [28], and the human cell differentiation networks [35]. The fractal network is characterized by a power-law scaling between the average distance and the number of nodes, as well as an organization of hubs which are preferentially connected to small degree nodes (disassortativity) rather than other hubs [33], [36].

### Dynamics and evolution of protein-protein interaction networks

The primary source of node evolution is assumed to be the duplication of single genes, groups of genes or whole genomes followed by divergence of duplicated genes [37]–[41], whereas link evolution has been modeled by different mechanisms such as random rewiring [42] and preferential attachment [31]. Network rewiring can for example be studied by tracking the evolution of network motifs after a whole-genome duplication event with subsequent divergence [37]. The change in protein-protein interactions between related species was shown to be substantially lower than the rate of protein sequence evolution [43]. These general considerations of network evolution indicated that frequently observed topological features like scale-free degree distribution (and preferential node attachment) are explained by mechanisms of network growth rather than by natural selection [42]. Later studies demonstrated that the evolutionary conservation and the topology of networks are readily explained by exponential duplication/divergence dynamics (DDD) [44], [45].

Mathematical models based on these mechanisms [45]–[49] often well reproduce the observed degree distribution 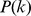 from numerical simulations of random graphs or analytical solutions of the asymptotic behaviors. However, two networks with the same 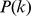 can have a totally different modular structure which is determined by higher-order correlations, and not captured by the simple degree distribution 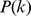. Furthermore, the simulated graphs generally do not correspond to the history of real networks, and the comparisons with experimental data are usually ambiguous as the parameters used in the models are difficult to measure directly.

Later studies utilize multiple approaches based on extant interaction networks for the explicit reconstruction of ancient networks which are then used to construct evolutionary arguments. Parsimony methods are motivated by the idea that network evolution is best explained by the least evolutionary changes [50], [51], whereas probabilistic methods reconstruct ancient networks of maximum likelihood [52], [53]. Integrating also phylogenetic information of the proteins represents their evolution more closely and therefore can further improve the accuracy of the reconstructed networks [54]–[56]. One of the most recent methods allows parsimonious reconstruction of multiple evolutionary events and at the same time it makes fewer assumptions compared to previous studies[51]. Dutkowski et al [56] suggested to use clusters of orthologous groups (COGs) to reconstruct ancestral proteins and ancestral interactions. Here we prefer the concept of COGs for reconstructing ancestral PPI network nodes, as it has been shown to be very robust and applicable even to evolutionarily distant genomes. COGs are therefore well established in comparative genomics (reviewed in [57]).

Most hitherto existing studies on network evolution were conducted on PPI networks of single organisms - mostly yeast, due to the rich amount of data - or on PPI networks of a small number of organisms. Integration of further organisms into evolutionary investigations allows for more general and more reliable statements on evolutionary principles. Facilitating the phylogenetic history of present-day proteins along with orthologous relationships between proteins offers a powerful possibility for the reconstruction of ancient proteins [58]. However, no similar concept exists for the inference of ancient interactions based on extant ones, therefore an underlying evolutionary model is necessary for their reconstruction.

The availability of large-scale PPI data for different species renders it now possible to study the dynamics of PPI networks of multiple species comprehensively by a novel approach combining advanced network theory and bioinformatics. Relying on the rich body of previous theoretical work as discussed above, we have established a theoretical framework by which we explicitly reconstruct and analyze ancestral PPI networks. The framework is based on clusters of orthologous groups for the genome-wide representation of ancestral proteomes on different taxonomic levels and a new stochastic model describing the duplication-divergence processes. The assumption of fractal topology of PPI networks, well justified by previous research, allows to properly handle the noisy and erroneous input data and to reduce the parameter space for the modeling of ancestral PPIs. The analysis of the degree distribution 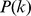 separates different species into two groups, characterized by a power-law (scale-free) distribution (*M. musculus*, *C. elegans*, *D. melanogaster* and *E. coli*), and an exponential distribution (*S. cerevisiae*, *H. sapiens* and *A. thaliana*). Irrespective of this, we find that their network topologies can be unified under the framework of scaling theory and characterized by a set of unique scaling exponents. The evolution of PPIs based on DDD can be modeled using two parameters, describing the probability for retaining an interaction after a duplication and the probability of a *de novo* creation of an interaction respectively. These iterative duplication events due to DDD imply a multiplicative growth of nodes, interactions and average path length that can be described by dynamic growth rates. The growth rates were obtained directly from the reconstructed networks. We observed that they are in agreement with the mechanisms of multiplicative growth, which was previously suggested in a theoretical study [33]. They are also in good agreement with the static measurements of the present-day networks.

## Results

### A uniform database allows for the comprehensive analysis of present-day interactomes

**Figure 1:**
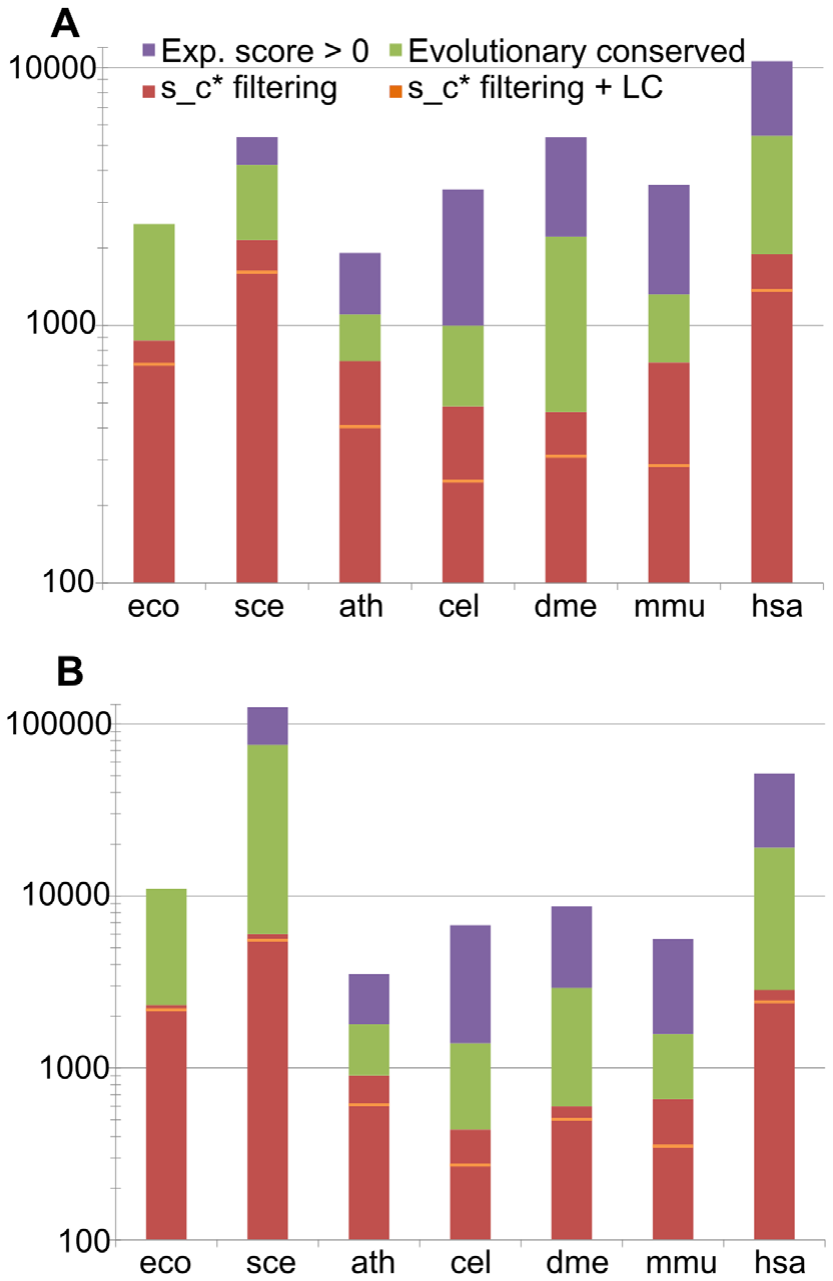
Input data overview. The numbers of proteins (nodes) and interactions extracted from STRING at each filter step before construction of the protein-protein interaction networks. Numbers are show on log-scale. (A) Number of nodes. (B) Number of interactions. Violet: STRING experimental score 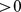, green: conserved on all evolutionary levels, red: after filtering at 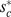, orange bars: after filtering at 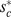 considering only largest (connected) component (LC); the largest component is necessary for the topological analysis.

To elucidate the broad principles governing the structure and the evolution of PPI networks, the most comprehensive and reliable data for as many species as possible are necessary. This is why the integrative database STRING [23] was chosen as the uniform source for physical protein-protein interactions. Besides functional interactions, which are not considered in this study, STRING provides physical PPIs for many species. For this study we selected seven species having the highest number of physical interactions in STRING and representing different lineages in eukaryotes and bacteria (Table 1). To construct high-quality physical PPI networks from these data, a number of filtering steps was performed. First, interactions without direct experimental evidence for the respective organism were removed from the analysis. This guaranteed that neither functional nor predicted physical interactions (interologs) were included in network construction. Second, proteins that are not contained in orthologous groups on all evolutionary levels defined by the eggNOG database [59] for the respective organism were excluded. This step removes all lineage specific proteins and provides consistent sets of nodes for the subsequent modeling of ancient PPI network (see below). Third, a threshold for confidence scores was introduced to separate high-confidence from low-confidence interactions, which were excluded from further analysis. The confidence scores are very differently distributed in the seven organisms of our study (Figure S1). Application of a uniform threshold score (e.g. 700) as generally suggested by STRING [23] would select very different fractions of the interaction data. As all further results of this study rely on the quality and unbiased selection of the interactions from STRING, we evaluated the effect of different score thresholds on the structure of the resulting networks. It is known that PPI networks are invariant or self-similar under a length-scale transformation [28]. This basic assumption about the structure of the resulting networks was therefore utilized to determine the optimal cutoff scores for each organism by three independent methods (see Materials and Methods, and Figure S2): percolation analysis, the Maximum Excluded Mass Burning (MEMB)[60] and the renormalization group approach [61]. The percolation analysis allowed to identify a point of percolation transition, at which a giant connected component first appears. This point of percolation transition was determined individually for each organism. At the point of percolation transition, the structure of the resulting networks changes from small-world to self-similar. The box-covering algorithm MEMB and the renormalization group approach served to validate the percolation analysis by confirming the self-similar structure of the resulting networks. Score thresholds between 400 (*A. thaliana*) and 980 (*S. cerevisiae*) were obtained for the different organisms (Figure S1 and Table 1). The filtering always removed the majority of proteins and interactions (Figure 1 and Table S1).

**Table 1:** Organism overview.

Organism name	Abbreviation	NCBI Taxonomy ID	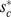	Nodes at 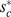	Interactions at 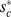
Escherichia coli K-12	eco	83333	440	873	2321
Saccharomyces cerevisiae	sce	4932	980	2144	6000
Arabidopsis thaliana	ath	3702	400	727	905
Caenorhabditis elegans	cel	6239	560	485	438
Drosophila melanogaster	dme	7227	700	461	598
Mus musculus	mmu	10090	700	718	658
Homo sapiens	hsa	9606	700	1891	2840

Overview of the organisms for which networks were reconstructed. For each organisms the scientific name, three-letter-abreviaton used in tables and figures, NCBI Taxonomy ID [71], filtering threshold 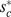, node count after filtering at 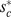 and interaction count after filtering at 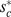 are shown.

For the topological characterization of the seven PPI networks we selected the largest connected component of every network. The application of the MEMB algorithm revealed a power-law relationship between the minimum number of boxes 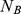 and the box diameter 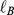 (Equation 1), which is typical for self-similar networks as shown in [60]. In this algorithm, 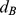 is the fractal dimension which characterizes the self-similarity between different topological scales of the network. It is known that the fractal dimension 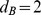 for random Erdös-Rényi (ER) network at percolation [62]. Our results suggest that the PPI networks have modular structures with correlated rather than random connections, since their values of 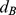 (Table 2) are different from the one predicted by the random percolation theory. Since the degree of modularity depends on the scale 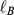, the modularity exponent 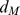 was calculated which can be used to compare the strength of modularity between dissimilar networks (Equation 7 and Figure S3). The degree of modularity of the networks ranges from low (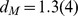) for *E. coli* and *S. cerevisiae* to high for *A. thaliana* (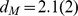), *M. musculus* and *H. sapiens* (both 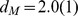) (Table 2). Since the trivial case of a regular lattice in 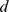 dimensions gives 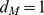, modularity exponents larger than one indicate a larger degree of modularity. Besides the fractality, another important topological measure is the distribution of degrees 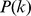. For many complex networks, 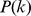 has a power law distribution with degree exponent 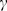 (Equation 2), which is characteristic of scale-free networks [31], [63]. On the other hand, if the equation describing the degree distribution becomes exponential (Equation 3), the network is said to have an exponential degree distribution (such as the ER graph [25]), indicating the existence of some typical scales for degrees [64]. Our results show that the PPI networks of different species are grouped into two categories with scale-free (*M. musculus*, *C. elegans*, *D. melanogaster* and *E. coli*) or exponential (*S. cerevisiae*, *H. sapiens* and *A. thaliana*) degree distributions (Table 2). The above two properties, the scale-invariant property and the degree distribution, can be related through scaling theory in a renormalization procedure [28]. At scale 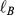, the degree of a hub 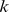 changes to the degree of its box 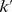 (Equation 4). A new exponent 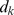 relates the fractal dimension 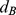 and the scale-free exponent 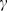, which states the fact that 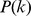 remains invariant under renormalization (Equation 5). The corresponding values obtained were consistent with our theoretical predictions, confirming the validity of our approach (Tabel 4).

**Table 2:** Scaling exponents (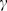, 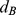, 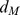) for the different species.

Species	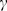	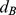	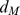	Scale-free	Exponential	Fractal
eco	1.9(1)	3.6(3)	1.3(4)	Yes	No	Yes
sce	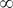	3.0(2)	1.5(1)	No	Yes	Yes
ath	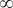	1.5(1)	2.1(2)	No	Yes	Yes
cel	2.6(1)	1.6(1)	1.8(2)	Yes	No	Yes
dme	3.0(1)	1.6(1)	1.3(2)	Yes	No	Yes
mmu	2.9(1)	1.7(1)	2.0(1)	Yes	No	Yes
hsa	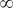	2.9(2)	2.0(1)	No	Yes	Yes

According to the values of the scaling exponents, the seven species listed are grouped into two categories: scale-free fractal networks and exponential (non-scale-free) fractal networks. The scale-free networks have a power-law degree distribution with exponent 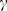, and the non-scale-free fractal networks have an exponential degree distribution with 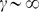. Notice that none of the networks are small-world. Instead, they are characterized by fractal/modular structures.

### The duplication-divergence model of network evolution enables the reconstruction of ancient interactomes

**Figure 2:**
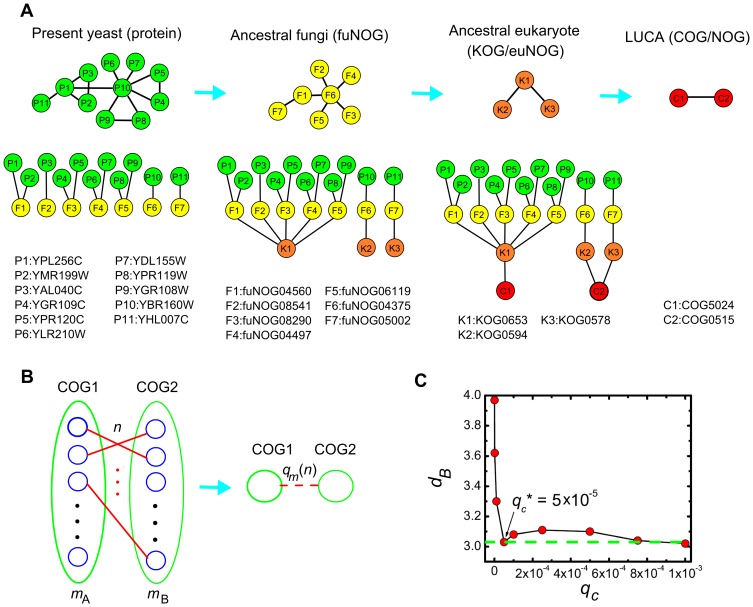
An example of the reconstruction process of the *S. cerevisiae* ancestral networks. (A) Illustration of the network reconstruction process. A subset of the empirical PPI network of *S. cerevisiae* is shown. The phylogenetic trees demonstrate how the proteins are grouped into COGs at different evolutionary levels. This information is used to identify the ancestral nodes. Note C2(COG0515) comprises other proteins which are not shown here. (B) The interaction between each pair of COGs is assigned a probability 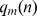 based on the duplication-divergence model. (C) The fractal dimension 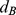 versus the cutoff 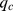 for the ancestral prokaryote network of yeast. By increasing 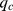, 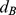 approaches to the value of the present-day network (dashed line). We choose cutoff 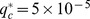 so that the ancestral network has the some fractal dimension as the present-day network. For 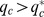, 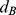 remains (approximately) as a constant.

According to the duplication divergence model, present-day PPI networks evolved from ancestor PPI networks through protein duplication and loss events followed by diversification of function and interactions. As the evolution of proteins can be well reconstructed using the concepts of orthology and paralogy, the **C**lusters of **O**rthologous **G**roups/ **N**onsupervised **O**rthologous **G**roups (COG/NOG) [65] assignments of all proteins were retrieved from the eggNOG 2.0 database [59]. Recent proteins were assigned to the NOGs of the most recent level according to the lineage of the organism and the taxonomic resolution of eggNOG 2.0. If multiple proteins were assigned to the same NOGs, duplication events have been reconstructed. This process was repeated between the NOG levels until the COG/NOG level, representing the last universal common ancestor (LUCA), has been reached. The NOGs on the different (evolutionary) levels represent the ancestral proteins at this evolutionary timepoint. Figure 2A shows an example of the reconstruction process for a subset of the ancestral networks of *S. cerevisiae*. The fuNOGs in Figure 2A (F1-F7) represent proteins in the ancestral fungi, KOGs/euNOGs (K1-K3) represent proteins in the ancestral eukaryotes and the COGs/NOGs (C1–C2) represent proteins in the LUCA. The two yeast proteins P1 and P2 which are assigned to F1 indicate a duplication of F1 in *S. cerevisiae*.

**Figure 3:**
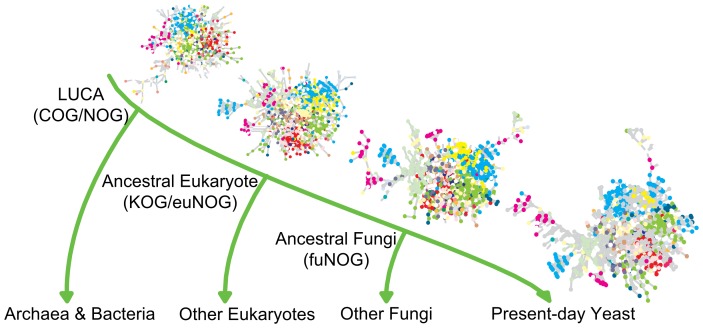
Ancestral networks that were reconstructed for the *S. cerevisiae* PPI network. Following the phylogenetic tree, PPI networks on different evolutionary levels were (re-)constructed: the present-day yeast (present-day protein), the ancestral fungi (fuNOG, last common ancestor of fungi), the ancestral eukaryote (KOG/euNOG, last common ancestor of animals, plants and fungi), and the Last Universal Common Ancestor (COG/NOG, last common ancestor of archaea, bacteria, and eukaryotes). The colors of nodes represent the different functional categories extracted from the eggNOG database [59].

While the ancestral nodes are obtained from the eggNOG database, the reconstruction of ancestral interactions is much more difficult. Although protein interactions are likely to be conserved between pairs of orthologs (``interologs"), the limited knowledge about recent interactions in many species and the link dynamics after duplications make it impossible to use this principle for the reconstruction of the links in ancient PPI networks. Thus, the most promising approach is to transfer interactions measured in today's PPI networks back in time, based on a model of link evolution. Here we applied the duplication divergence model (see Materials and Methods) to estimate the probability of the ancient interactions based on today's PPI networks. A probability is assigned to the interaction between each pair of COGs/NOGs (representing ancient proteins) based on the number of possible interactions between proteins in both COGs/NOGs and the number of actually observed interactions in the present-day networks (Figure 2B). The parameters required for the model are derived by a fitting approach, so that the properties of the resulting ancient networks resemble those of today's PPI networks. We assume that general properties of PPI networks are constant during evolution (Figure 2C). The reconstruction is additionally constrained by the underlying reconstruction of the ancient proteins. The parameters defining which interactions are transferred back in time are the fraction of interacting pairs in the ancestral network at time 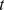, 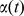, the probability 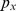 that an interaction is retained after a duplication and the probability 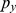 that a new interaction is created *de novo*. An overview of the fitted parameters for all organisms is shown in Table 3. We observed that 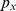 values range between 0.5 and 0.7, but 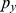 values are multiple orders of magnitude smaller. These parameters indicate that link evolution after duplication is the rule and de-novo creation is the exception. The values are in good agreement with results from an earlier study on *S. cerevisiae* [32]. A schematic representation of the reconstruction of the ancestral networks is given in Figure 3, which shows the networks at the evolutionary levels that were reconstructed for *S. cerevisiae*.

**Table 3:** Fitting parameters in the duplication-divergence model for all organisms.

Species	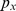	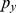	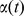								
			prNOG	roNOG	maNOG	veNOG	inNOG	meNOG	fuNOG	KOG/euNOG	COG/NOG
eco	0.7	0.0008									0.007
sce	0.7	0.0002							0.0008	0.0007	0.001
ath	0.7	0.0001								0.003	0.008
cel	0.5	0.0004						0.002		0.001	0.005
dme	0.5	0.0004					0.003	0.004		0.004	0.004
mmu	0.7	0.0002		0.001	0.001	0.001		0.001		0.001	0.003
hsa	0.7	0.0002	0.0002		0.0004	0.0005		0.0005		0.0003	0.0004

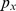
 and 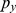 are time-independent and describe the probability that an interaction is retained after a duplication and the probability that an interaction is created de novo, respectively. The fraction of interacting pairs in the ancestral network at time 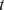 is represented by 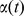. There are in total nine ancestral time levels for the organisms investigated: the ancestral primates (prNOG), the ancestral rodents (roNOG), the ancestral mammals (maNOG), the ancestral vertebrates (veNOG), the ancestral insects (inNOG), the ancestral animals (meNOG), the ancestral fungi (fuNOG), the ancestral eukaryotes (KOG/euNOG), and the LUCA (COG/NOG). Existing time levels are specific for every species depending on its lineage.

The consistency of the ancient PPI network was investigated by calculating their pair-wise overlaps. Therefore, the numbers of overlapping nodes and interactions between the organisms on all evolutionary levels were obtained (Figure S4). *S. cerevisiae* has a relatively large overlap with all other species due to its network size, which is the largest of all organisms considered in the study. Whereas *H. sapiens* shows relatively large overlaps with all other organisms, the highest overlap is, as expected, with *M. musculus*, which is evolutionary most closely related to *H. sapiens*. *E. coli*, which has the third largest network of the organisms, exhibits small overlaps to all other organisms, except for *S. cerevisiae*, which is the only other unicellular organism among the organisms of this study.

### The change of interactome structures over time is explained by multiplicative growth mechanisms

The reconstructed ancestral PPI network represent a series of snapshots in the evolution of the present-day networks of the respective species. By measuring the structural features of the networks at these different time points, the growth principles of the PPI network can be studied. Our results suggest a multiplicative growth mechanism (see Materials and Methods) as proposed in Ref. [33].

**Figure 4:**
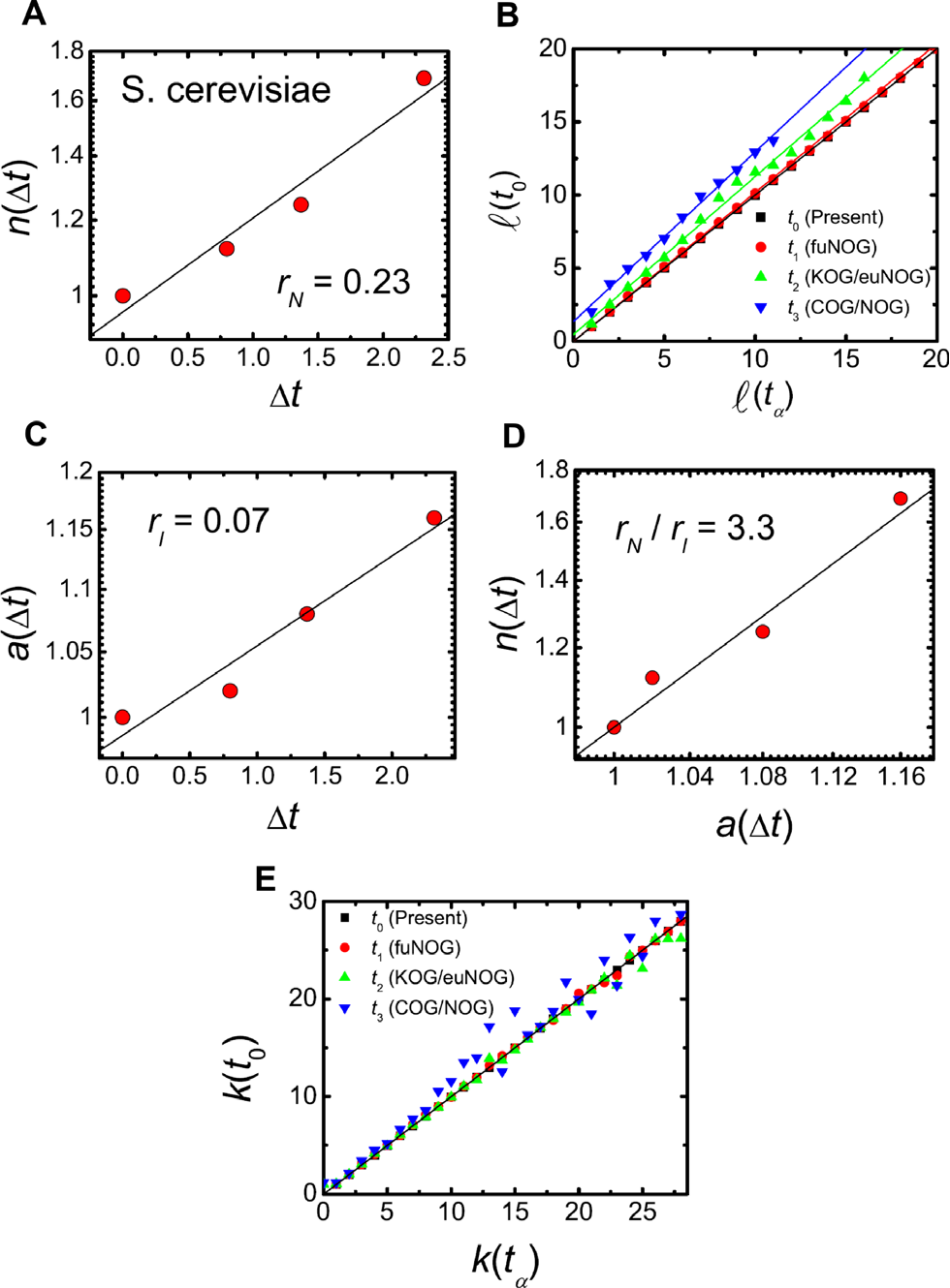
Multiplicative growth mechanism of the *S. cerevisiae* PPI network. (A) Semi-log plot of 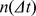 vs. 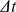. The growth rate  is obtained from a linear fitting. The unit of time is Gyr. (B) Scaling between 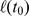 and 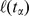. Each point is an average over many pairs of nodes in the network with the same 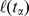. The slope of the linear fitting gives 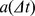, where 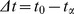 is the time difference between two evolutionary levels. (C) Semi-log plot of 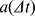 vs. 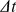. The growth rate 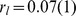 is obtained from a linear fitting. (D) Log-log plot of 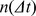 vs. a(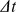). The scaling shows that the ratio between two growth rates (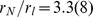), is close to the static measure of the fractal dimension 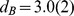. This confirms the relationship Equation (16). (E) Scaling between 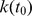 and 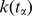. Each point is an average over many nodes with the same 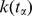. Large degrees (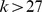) are not included in this plot since there is not enough number of samples to make meaningful statistics. The slope of the linear fitting gives 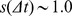, which is consistent with an exponential degree distribution.

We first studied the PPI networks *S. cerevisiae*, which is the largest network in our analysis. Figure 4A shows that the time-dependent generator 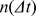, as well as the number of nodes 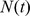 (see Equations 13 and 14), follows an exponential form with the nodes growth rate 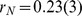/Gyr. The linear scaling between 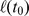 (the distance between two present-day proteins) and 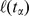 (the distance between two corresponding COGs/NOGs at time 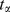) on all evolutionary levels is shown in Figure 4B. The growth rate of the distances is found to be 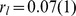/Gyr for the *S. cerevisiae* network (Figure 4C). The two growth rates satisfy the condition 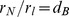 (Figure 4D and Table 4). The result relates the dynamic growth rates 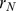 and 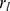, to the static exponents 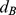. This means that the nodes and distances do not grow independently but they grow at rates with a fixed ratio which is equal to the fractal dimension 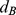 and therefore conserve the fractal structure rather than becoming small-world. The linear scaling between 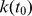 (the degree in the present-day network) and 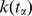 (the degree of the corresponding COG/NOG at time 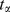) is shown in Figure 4E. The growth rate for the interactions 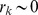 was found for *S. cerevisiae*, which suggests 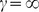 according to Equation (19). This implies that the *S. cerevisiae* network has an exponential degree distribution, which is consistent with the direct observation of the static network structures (Table 4 and Figure S5). While the multiplicative growth was originally proposed as a growth mechanism of nodes, distances and degrees [33], simple generalization of the same mechanism could be used to predict the growth rate of modularity (Equation 21 and 22). For example, it was found that 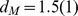 and 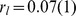/Gyr, Equation (22) predicts 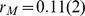/Gyr. This assumes that the exponent 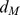 is invariant, although the modules might involve with time.

**Table 4:** Scaling exponents, growth rates and their relationships.

	static exponents				dynamic growth rates					
Species	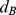	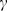	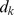	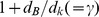	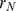	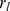	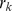	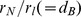	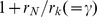	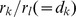
eco	3.6(3)	1.9(1)	3.3(4)	2.1(1)	0.06	0.02	0.07	3	1.9	3.5
sce	3.0(2)	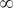	0.0(1)	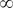	0.23(3)	0.07(1)	0.0(1)	3.3(8)	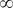	0
mmus	1.7(1)	2.9(1)	0.8(1)	3.1(4)	0.22(3)	0.15(1)	0.14(2)	1.5(3)	2.6(4)	0.9(2)
hsa	2.9(2)	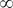	0.0(2)	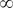	0.23(2)	0.08(1)	0.0(1)	2.9(5)	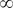	0

Scaling exponents (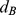, 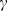, 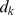), growth rates (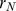, 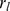, 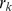) and their relationships derived from the dynamic analysis (The growth rates of *E. coli* do not have uncertainties because there are only two time levels). Here we selected the three largest networks (*E. coli*, *S. cerevisiae*, and *H. sapiens*) and one sample (*M. musculus*) representing the smaller networks.

**Figure 5:**
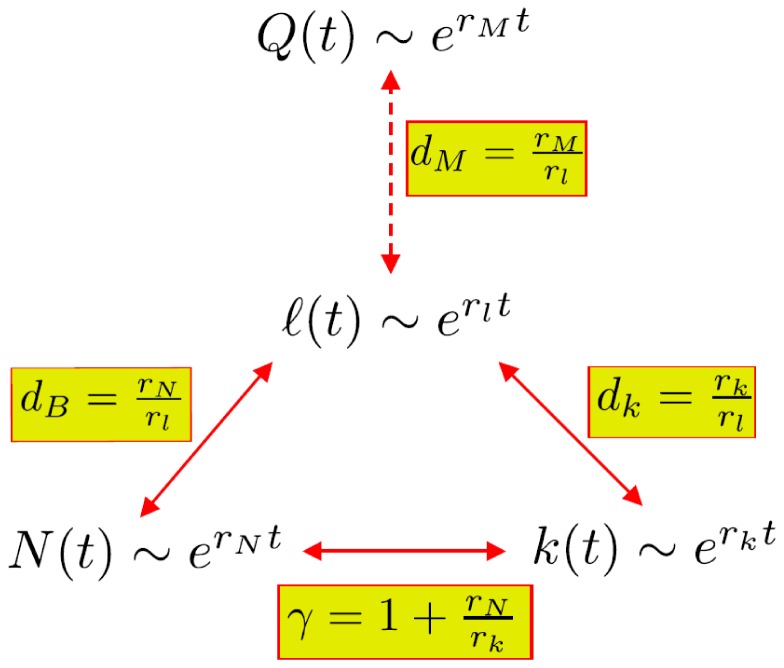
Summary of the evolutionary mechanism. Conservative and multiplicative laws determine the static scaling exponents (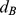, 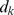, 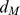, 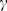) in terms of growth rates (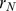, 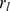, 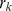, 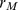). The three theoretical predictions (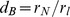, 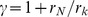, and 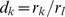) have been corroborated by empirical calculations, while the remaining relation 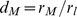 is a prediction open for test.

For studying the growth mechanisms in the PPI network of other species, we selected the two further larger networks (*E. coli* and *H. sapiens*) and one PPI network representing the smaller networks (*M. musculus*). We observed multiplicative growth mechanisms also for these three PPI networks (Table 4 and Figures S6, S7 and S8), indicating that these growth principles are species-independent and thus universal. Furthermore, the degree exponents, fractal dimensions and the modularities obtained from this dynamic analysis were found in very good agreement with those from the static analysis described above (Table 4). Our results confirm the proposed relationship between the static scaling exponents and the dynamic growth rates (Figure 5). The core of the results are the exponential growth of the system quantities (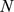, 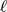, 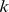, 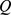), the relations between the static exponents (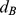, 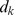, 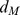, 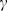) and the dynamic rates (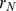, 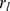, 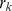, 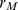) (see Materials and Methods for a detailed explanation).

## Discussion

The evolution of protein interaction networks is much less studied compared to e.g. the evolution of DNA and aminoacid sequences. This is not only a consequence of our sparse data on PPI networks, as experimental approaches have intrinsic limitations and genome-wide screens are very costly. Complete PPI networks, considering then entire networks of protein-protein interactions across all possible environmental conditions and developmental stages, are far from being characterized even for unicellular model organisms such as *E. coli* or *S. cerevisiae*. There are also a number of conceptual questions how to study the evolution of networks. On which levels are biological functions relevant for the evolution of a PPI network (e.g. on the levels of binary interactions, protein complexes, functional modules or entire networks)? How are the emergent features of a PPI network selected in evolution (e.g. robustness and stability)? How is the evolution of PPI networks connected with other types of molecular networks? Most of these questions could hardly be answered until now. Here we focus on one of the most basic problems in PPI network evolution: what are the universal dynamic principles by which PPI networks grow and change over time? The increasing amount of PPI data for different organisms as well as orthology reconstruction on different taxonomic levels allowed us to investigate the network topology and growth of multiple present-day and presumed ancient organisms in this study.

### The structure of present-day PPI networks from multiple species

Ideally, complete PPI networks from multiple species would have been used for this study. Due to the limitations in the experimental determination of PPI, no such data are so far available. Therefore we had to compile a representative set of input PPI networks from the heterogeneous, incomplete and erroneous PPI data available. Although the integrative STRING database very much simplified this task by providing the PPI data from multiple organisms in a unified database scheme, the distribution of experimental interaction scores was very different among the selected species. This might result from different experimental strategies, but makes the filtering by a static score threshold questionable. For our study we expected the present-day PPI networks to represent interactions of comparable strength and confidence. A novel filtering approach based on the assumption of self-similar topology was therefore implemented for the filtering of the initial PPI data from the STRING database. We solved the problem by applying a percolation analysis, which is based on the idea of strength of links inspired from sociology, and has been recently used to define functional brain networks from fMRI signals [66]. The percolation theory unambiguously defines the critical threshold for the ranked scores in the STRING database, which separates the small-world from the large-world of self-similar structures: above or at the critical connectivity, strong links form a highly modular, large-world fractal backbone, and below the critical connectivity, weak ties establish shortcuts between modules converting it to a small-world network [66], [67]. The resulting score thresholds varied significantly between the species. Considering the scoring scheme of the STRING database, this might be explained by varying proportions of individual vs. high-throughput experiments in the database. However, in all networks a major fraction of the interactions was removed through the filtering. The remaining PPI are expected to form representative (as defined by network topology) interaction networks on a species-specific confidence level. Remarkably, a significant fraction of nodes was removed as they were not represented on all taxonomic levels of clusters of orthologous groups in the eggNOG database. This phenomenon is not only present in the version 2.0 of this database, but to a different extent also in the new version 3.0. Besides technical reasons it might also be caused by complex evolutionary histories (e.g. due to horizontal gene transfer) in protein families. The filtered PPI networks in our study therefore contain only proteins with a clearly traceable, mainly vertical evolution. The success of the filtering operations can not be directly assessed, as no additional gold-standard PPI data are available. However we observed that structural and topological properties of the filtered PPI networks were comparable also beyond the initial assumption of self-similarity, indicating that these data are a reasonable basis for further analysis in this study.

### Reconstructing ancient PPI networks based on the duplication-divergence model

The duplication-divergence mechanism has been proposed by numerous previous studies for the dynamic growth of PPI networks. Phenomena like preferential attachment and correlation of evolutionary rate vs. degree in PPI networks might be consequences of this growth rules. To challenge this theory we developed an algorithm for the reconstruction of ancient PPI networks based on present-day data. Although the parameters of the duplication-divergence model might be variable in evolutionary time, the limited data available make only a general estimation possible. The duplication-divergence model comprises two fundamental components: gene duplications and link dynamics. The evolution of genes has been directly reconstructed from clusters of orthologous groups. As these clusters are widely used in bioinformatics e.g. for prediction of gene function, the node structure of the ancient networks can be considered to be very authentic. However, it embodies only a fraction of the ancient proteomes. Proteins without present-day interactions and proteins removed during the initial filtering are missing, as well as proteins that have been lost in the evolution of the species selected for this study. The ancient nodes therefore specifically represent the ancestors of the nodes in the present-day PPI networks.

Because the link dynamics are so far inaccessible by any orthology-driven approach, we developed an algorithm to reconstruct the most probable ancestral interactions based on the stochastic duplication-divergence model. The fitting parameters in this model were determined from the COG data, which are independent of the network topology. As sequences of genes, interactions are mainly created through gene duplication. However, previous studies did not agree whether it is more likely to retain or to lose an interaction after gene duplication [32], [37], [68]. In contrast to the evolution of sequences, *de novo* gain of interactions are expected to occur much more frequent than the *de novo* formation of genes. This complicates the reconstruction of ancestral interactions significantly. Here we have developed a solution of this problem based on a novel stochastic model of duplication/divergence constrained by the node structure (COG/NOG based) and the assumption of self-similar topology for the determination of the interaction probability cutoffs. As expected, Table 3 suggests for all species that the probability to retain an old interaction is equal or higher (0.5–0.7) than that to lose an interaction, and is several orders higher than that to gain a new interaction (0.0001–0.0008). That is, 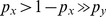. This means that the majority of present-day interactions are inherited from ancestral interactions, while the generation of new interactions is much less frequent. A comparison of our results to values from earlier studies on *S. cerevisiae* [32], [37], [68] indicates very similar size ranges for the probability for retaining an interaction after a duplication and the probability for creating a new interaction *de novo*. The good agreement between our results and results from earlier studies, conducted on different datasets using different approaches, further supports the duplication divergence model of network evolution.

While it is known that the duplication-divergence model results in an exponential growth of the network size [45], there is no simple analytical way to predict the dynamics of distance and modularity based on the model. However, it is important to note the connections between the network dynamics and the parameters in the duplication-divergence model. For example, if 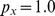, the distances between proteins remain the same (Figure S9C) after duplications, while the number of proteins grows exponentially. This results in a network of small-world structure and exponential dynamics, which shows that the duplication-divergence process does not necessary imply the fractality and the multiplicative growth. When 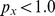 as observed in Table 3, there is a probability that an old interaction is deleted, and the new protein is connected to the old protein through a longer path (Figure S9C). This increases the distances between proteins. In fact, based on direct measurements of the reconstructed networks, we found multiplicative (exponential) growth of distances. The multiplicative growth of both, nodes and distances, conserves the fractal/modular structure rather than becoming small-world.

A direct evaluation of the results is impossible as independent data on ancient PPI networks is unavailable. However, the consideration of different species in this study enables an indirect assessment of our modeling results. Ideally, if the initial present-day PPI networks would be complete and free of errors, they should result in equivalent networks on the ancient taxonomic levels. E.g, the present-day *H. sapiens* and *M. musculus* networks should predict the same ancient networks for the ancestral mammal, the ancestral vertebrate etc. Assessing the pairwise similarities between the ancient PPI networks, we observed partial overlaps corresponding to the size of the present day networks (representing completeness) and also according to the lifestyle and evolutionary distance of the organism. These results support the validity of the reconstruction algorithm based on the duplication-divergence model, but they also indicate the substantial limitations of the present-day PPI data.

Despite the strong evidence for the duplication-divergence model, the possibility of a model-dependent bias may still remain. The model favors a multiplicative growth rather than a linear growth over a relatively wide range of parameters. Further studies are required to test whether this preference is a biological consequence, or induced by the choice of the model. On the other hand, there exist other models [69] consistent with a multiplicative growth. However, these models generally have no relevance to biological evolution, and therefore are not used in the study of PPI network evolution.

### Universal dynamic principles determine the growth of PPI networks

The explicit reconstruction of ancestral PPI networks for 7 selected species provides the unique opportunity to study their growth dynamics. Although the filtering of initial PPI data and the reconstruction algorithm utilize assumptions of fractal topology, they do not necessarily result from multiplicative growth. This means, whereas multiplicative growth implies fractal topology, other growth mechanisms might produce fractal networks as well, such as for instance a pure percolation process on the network [70]. Therefore we analyzed the growth of number of nodes, number of edges, size and modularity of the networks over time for the three larger networks and one selected smaller network. In all networks we found a very good agreement between the multiplicative growth principle and the observations in the present-day and ancient PPI networks. Furthermore we found an excellent matching between the results from static and dynamic analysis, which are independent approaches. These results support both the duplication-divergence model and multiplicative growth as fundamental mechanisms in the long-term dynamics of PPI networks.

Our approach allowed to determine the network topologies of multiple present-day and presumed ancient organisms based on two widely used databases - STRING, providing information about functional and physical protein interactions, and eggNOG, providing information about the evolutionary relationships of proteins. To our knowledge, such an extensive characterization of multiple extant and ancient networks has not been performed until now, as it is important for formulating and verifying mathematical models describing the evolution of protein networks. The network properties determined from topological network analysis correspond well to the properties determined from dynamic analysis based on the duplication-divergence evolutionary model. This provides strong evidence for the correctness and the universality of the proposed mathematical model of network dynamics and evolution.

## Materials and Methods

### Databases

A database dump of the STRING database (release 8.3) was downloaded from ftp://string-db.org/ and a local database copy was set up. Binary protein interactions for the studied organisms [71] (Table 1) with experimental scores above zero were extracted to obtain experimentally confirmed physical interactions. The eggNOG database (release 2.0, ftp://eggnog.embl.de/eggNOG/2.0/) was used to obtain the assignment of proteins to clusters of orthologous groups (COGs/NOGs) on different taxonomic levels. These levels are species-specific and defined in the eggNOG database. There are in total nine ancestral time levels for the organisms investigated: the ancestral primates (prNOG), the ancestral rodents (roNOG), the ancestral mammals (maNOG), the ancestral vertebrates (veNOG), the ancestral insects (inNOG), the ancestral animals (meNOG), the ancestral fungi (fuNOG), the ancestral eukaryotes (KOG/euNOG), and the LUCA (COG/NOG). Figure 3 exemplifies the ancestral time levels for *S. cerevisiae*. In the initial filtering only proteins that were conserved on all evolutionary levels defined for the respective species were considered, thus every protein had an assignment to all its evolutionary levels. Our reconstruction algorithm and reconstructed networks are available at http://fileshare.csb.univie.ac.at/ppi_evolution_pone2013.

### Reconstruction of the filtered present-day protein interaction networks

The STRING confidence scores were used to assess the reliability of the protein-protein interactions. For the identification of the score threshold for reliable interactions the finding of Song et al [28] that PPI networks are scale-invariant and self-similar was taken as a basis. A threshold score 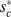 above which interactions were deemed reliable was determined and confirmed for each organism by the following three independent methods:

- Percolation analysis. 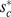 can be found as the threshold of a percolation transition of the network. When networks are reconstructed for all possible confidence scores, the percolation threshold 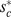 represents the first jump in the size of the largest cluster, while the size of the second largest cluster peaks at this point (see Figure S2A). The percolated cluster, also called giant connected component, is formed by links whose confidence score is higher or equal to 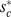. We observed a series of jumps in the percolation process, which suggests a multiplicity of percolation transitions [66], [72]. This is different from a random percolation (Figure S2A inset), where only single transition point exists. Our results show that the percolation process of PPI networks is more complicated than a simple uncorrelated percolation process, due to the modular organization and the strong correlations between protein interactions.
- MEMB-algorithm. The box-covering algorithm MEMB [60] (Figure S2B) was used to tile the network with the minimum number of boxes 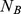 of a given box diameter 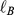. 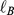 was defined such that the maximum distance in a box is smaller than 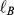, and distance was measured as the number of links on the shortest path between two proteins. A power-law scaling of 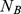 and 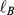 at 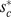 confirms the fractality of the network at the percolation threshold (Figure S2C).
- Renormalization group analysis. The renormalization group approach [61] was used for another confirmation of the 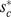 threshold as the transition point between small-world and fractal phases. The renormalized network is built by replacing the boxes by ``supernodes" and two supernodes are connected if there is at least one link between two nodes in their respective boxes. The relationship between the average degree of the renormalized network, 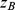, and the average number of nodes in each box 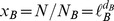 gives information about whether the network is small-world (positive slope), fractal (negative slope) or at the phase transition 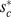 (slope of 0) (see Figure S2D).

The addition of links of scores below 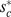 (defined from percolation analysis, Figure S2A) converts a fractal network (above 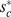) into a small-world network. That is, the power-law relation (Equation 1) transforms into an exponential decay characteristic of small-world (MEMB-algorithm, Figure S2C), and the slopes become positive in Figure S2D (renormalization group analysis). Therefore, the three independent methods are consistent with each other. From the resulting networks, the largest connected component at 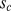
* was used for topological analysis.

### Topological properties of the networks

The fractal dimension 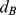 was measured from the MEMB algorithm, by fitting the relationship between the minimum number of boxes 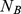 and the box diameter 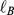 to a power-law function [28] (see Figure S2C for *S. cerevisiae* and *M. musculus*):
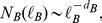
(1)where 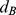 is the fractal dimension which characterizes the self-similarity between different topological scales of the network. The values of 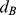 for all species are summarized in Table 2.

The degree distribution 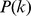 was measured and the degree exponents 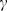 [31] were determined. For some networks (*M. musculus*, *C. elegans*, *D. melanogaster* and *E. coli*) it was shown to follow a power law distribution with degree exponent 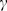:
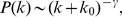
(2)where 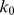 is a small cutoff degree. For others (*S. cerevisiae*, *H. sapiens* and *A. thaliana*) the parameters became 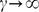, 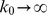 with fixed 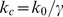 and the equation had an exponential form:
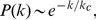
(3)
Figure S5 shows 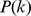 of two species, *S. cerevisiae* (exponential) and *M. musculus* (scale-free), which are characteristic of the behaviors found across all species. Table 2 summarizes the values of 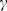 for all the species.

The above two properties, the scale-invariant property, Equation (1), and the degree distribution, Equation (2), can be related through scaling theory in a renormalization procedure[28]. At scale 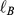, the degree of a hub 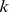 changes to the degree of its box 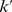, through the relation:
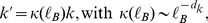
(4)A new exponent 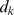 relates the fractal dimension 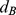 and the scale-free exponent 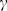 through
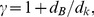
(5)which states the fact that 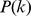 remains invariant under renormalization. For the *S. cerevisiae* PPI network, we found 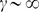, 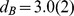, and 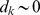, and for the *M. musculus* PPI network, we found 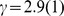, 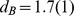, and 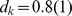 (Figure S10). The values of 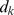 are summarized in Table 4. The results are consistent with our theoretical prediction, Equation (5).

### Modularity

The modular organization [35], [66], [73] of the network was investigated by the analysis of the links inside and between topological modules. Modules were defined by the boxes detected by MEMB algorithm. To capture the degree of modularity of the network, the modularity ratio 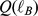 was defined as a function of the size of the modules, 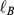:
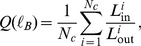
(6)where 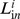 is the number of links between nodes inside the module 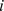, 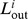 is the number of links from module 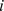 connecting to other modules and 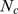 is the number of modules needed to tile the network for given size 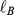. Large values of 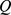 correspond to a structure where the modules are well separated and therefore to a higher degree of modularity. The degree of modularity depends on the scale as:
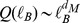
(7)which defines the modularity exponent (see Figure S3).

### Construction of the ancient protein interaction networks

The reconstruction of the ancient networks is based upon two integral parts: the identification of the ancestral proteins due to their evolutionary relationships and their assignment to COGs/NOGs (described above) and a duplication-divergence model describing the link dynamics during evolution. A fundamental assumption for both parts is that the structural network features are time-invariant.

The ancestral nodes were obtained from the assignment of present-day proteins to COGs/NOGs provided by the eggNOG database on different time levels.

The next crucial step was to decide when to transfer present-day interactions to the presumptive ancient network. Each COG could comprise several proteins, and the proteins in the same COG pair may or may not interact. Rather than transferring every present-day interaction, it is necessary to assess the probability that the respective COGs interact. For example, if two COGs comprise 10 proteins each, but there is only one interaction (out of 100 total possible interactions) between these proteins in the present-day network, it is improbable that these COGs (or the ancient proteins they represent) actually interacted.

In order to estimate this probability, the relationship between the number of total possible interactions and the number of actual interactions between the proteins which participate in these COGs is considered. As illustrated in Figure 2B, if two COGs A and B comprise 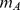 and 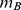 proteins each, then there are 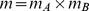 total possible interactions between the proteins in the COGs. Out of the 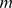 possible interactions, let 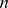 be the number of interactions that are actually detected in the present-day experimental data. One simple way is to assume the ancestral link probability between COGs A an B is proportional to 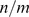. However, this assumption is oversimplified, since this probability does not only depend on the ratio 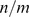, but also on the value of 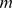. For example, depending on the data it is 10 times more probable to find 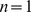 actual interaction out of 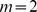 total possible ones, than to find 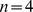 actual interactions out of 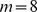 possible ones, although they have equal ratio 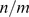.

In the reconstruction method, a probability 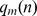 (see below how 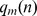 is calculated) is assigned to the ancestral interaction between the two COGs. The value of 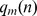 is calculated from a stochastic model described below. This way, a network of COG-COG interactions with weighted edges given by 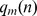 is constructed, where the edges with large weights are regarded as the most-likely interactions constituting the ancestral network.

The final step is to determine a proper cutoff of 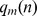 since COG pairs with low 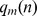 would most probably not interact. Only interactions with probability higher than 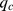 (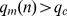) are included in the analysis. Changing this cutoff value allows to switch the sensitivity or selectivity of the ancestral interactions. To determine the cutoff, it is required that the reconstructed networks at different time levels have invariant topological features. In practice, the fractal dimension 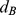 in each ancestral network is measured explicitly as a function of the cutoff 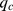 (Figure 2C), and a critical value of 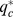 is determined when 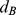 reaches to the same value as the present network. For example, in the case of the *S. cerevisiae*, we find 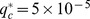.

In order to estimate the probability of the ancestral interactions 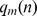, we developed a symmetric stochastic evolution model of the protein interaction network based on duplication-divergence processes [38]–[41]. The model takes into account the deletion of duplication-derived interactions and *de novo* creation of interactions. An analytical function of link probability is derived to compare with experimental data and determine the parameters.

Based on the mechanism of genomic duplication and divergence two general modes are considered: (i) Mode I (Figure S9A): protein A initially interacts with protein B, and protein A is duplicated into two proteins A and A′. The duplicated proteins A and A′ have equal probability 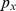 to copy the interaction link with protein B. (ii) Mode II (Figure S9B): protein A and B do not interact with each other initially. There is a probability 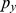 that the duplicated proteins A or A′ gains a new interaction with protein B.

The evolution of the network is completely specified by the parameters 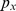, 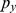 and its initial condition. 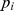 describes the probability of an interaction between any pair of new proteins after 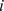 total duplications (protein A and B duplicates 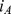 and 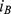 times each, and 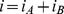). Two successive duplication steps can be represented by the recursive relation of 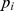


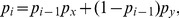
(8) where the first term comes from the contribution of the existing link at 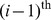 step, and the second term is from the non-existing link. Equation (8) can be solved recursively, producing a formula of 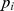 which only depends on 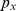, 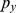 and the initial condition:
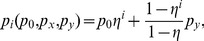
(9) where 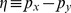. Here 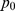 describes the initial condition: 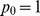 if the pair of proteins initially interact with each other, otherwise, 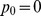.

After 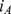 (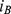) duplications, the initial protein A (B) evolves into a cluster comprising 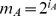 (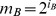) present-day proteins. 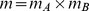 is the total number of possible interactions, and 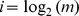 is the total number of duplications (Figure S9B). Let 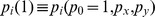, and 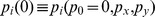. For a pair of clusters with 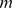 total possible interactions, the probability 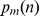 that 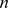 pairs of these proteins actually interact, given that each pair have independent probability 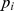, is represented by a binomial distribution. If the initial pair of ancestral proteins interact, then 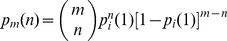; if they do not initially interact, then 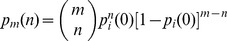. 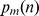 of a network is a combination of these two cases. Assume that 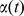 is the fraction of interacting pairs out of total possible pairs in the ancestral network at time 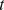. 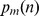 can be calculated as:
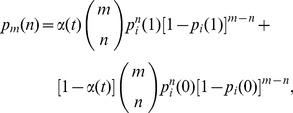
(10)The first term describes the interacting pairs in the ancestral network, and the second term is from the non-interacting pairs. Note that 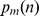 depends on time 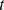 since we assumed that 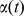 could be different at different time levels.

Equation (10) depends on three parameters 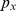, 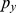 and 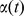 for each time 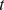. It was assumed that 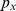 and 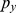 are constants at different time levels, and 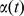 is time-dependent. To determine these parameters, 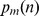 is fitted to the values derived from the present-day networks and COG data. For each evolutionary level 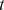, we first found the number of possible COG pairs that contains 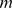 total possible interactions, 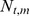. Out of 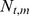 total pairs, we counted the number of COG pairs that have 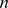 actual interactions, 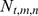.

Statistically, the ratio 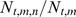 should represent the probability 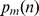. In order to find the best fitting, we minimized the objective function
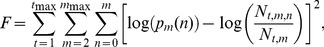
(11)where 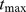 is the maximum time level, and 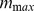 is the maximum 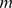 used in fitting. Our objective function is very similar to the standard residual sum of squares (RSS). The logarithm values are used here because 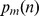 has an exponential behavior (Figure S11). Minimization of Equation (11) is an unconstrained nonlinear optimization problem on multiple parameters, which was handled by the function *fminsearch* in MATLAB R2012a.


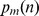
 was fitted to the measured values for all organisms. To have meaningful sample sizes, 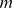 was restricted to be between 2 and 8. Figure S11 shows the results of three species: *S. cerevisiae*, *M. musculus*, and *H. sapiens*. The fitted curves are in good agreement with empirical data. The fitted parameters for all species are summarized in Table 3.

Since 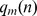 is the probability to have an ancestral link for a given 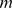 and 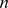, it is proportional to 
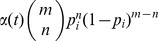
, which is the first term in Equation (10). With a proper normalization, we obtained:
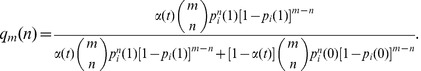
(12)


Equation (12) was used to reconstruct the ancestral networks (see Figure 2B) with fitted parameters from Table 3.

### Determination of the growth principles

To determine the dynamical processes governing the changes in network structures over time, the growth rates of nodes, distances and degrees were empirically determined. In detail, the following values were determined directly from the networks at each timepoint 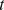: the number of nodes 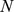, the number of links 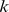, the distance 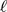 between two COGs. Our results support the multiplicative mechanism proposed in [33] to account for the fractal, modular and scale-free nature of PPI network structures.

The determined growth rates were set in relation to the scaling exponents of the networks, which were obtained from the static topological network analysis. Estimations for the divergence times between the organisms were derived from [74] and are listed in Table S2, which provide the time 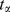 representing the time levels of COGs/NOGs.

The increase in the number of nodes over time is best approximated by an exponential function:
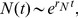
(13) with a growth rate of the number of nodes 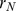. This implies the multiplicative growth form of 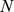 with a time-dependent generator 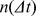:
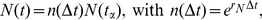
(14) where 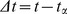. Figure 4A and Figure S6A show this growth mechanism for *S. cerevisiae* and *H. sapiens*. Table 4 summarizes measured 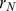 of all species.

Next, we consider the distance between two COGs in an ancestral network, 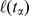, and compare with the corresponding distance 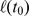 in the present network. 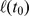 is measured as the distance between the two hubs in each COG, where a hub is the protein with maximum degree inside each COG. If two hubs have the same degree, then the average value was taken. The evolution of distance 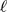 can be modeled by a similar form:
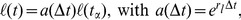
(15)


This suggests an exponential growth of distances instead of a linear growth. The multiplicative growth of 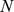 and 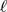 is consistent with the fractal scaling law Equation (1). On the contrary, a combination of exponential growth of nodes and linear growth of distances would result in an exponential scaling between nodes and distances, which represents a small-world network [33]. Figures 4B, S6B, S7A, and S8A show the linear scalings between 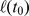 (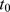 is the present time) and 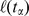 for four representative species, *S. cerevisiae*, *H. sapiens*, *M. musculus*, and *E. coli*. 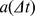 was obtained by liner fittings and was used to calculate the growth rates 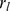 (see Figure 4C for *S. cerevisiae* and Figure S6C for *H. sapiens*). The values of 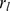 of all species are listed in Table 4.

The growth Equations (14) and (15) can be combined to obtain a power-law relation between the distances and the number of proteins with an exponent 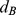 given by the ratio of the growth rates,
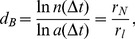
(16)


Equation (16) shows the relation between the static exponent 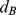 and dynamic growth rates 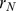 and 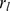. This theoretical prediction is tested in Figures 4D, S6D, S7B, and S8B, which confirm a power-law relation between 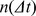 and 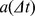. Table 4 shows that 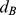 measured from static network structure is in good agreement with the value 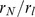 predicted from dynamic growth rates.

The number of interactions 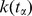 of each COG at time 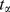 was compared with the degree 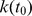 in the present yeast network, where 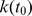 was the degree of the hub in each COG. Our results (Figures 4E, S6E, S7C, and S8C) show that the number of interactions 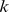 also follows a general form of multiplicative growth with a time-independent generator 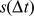:
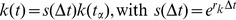
(17)



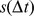
 was measured from linear fitting of this scaling between 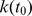 and 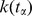. The growth rates 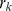 were measured and listed in Table 4. In particular, for networks of exponential degree distributions (such as *S. cerevisiae*, *H. sapiens* and *A. thaliana*), 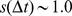 and 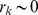 (see Figure 4E for *S. cerevisiae* and Figure S6E for *H. sapiens*), which suggests that the degrees are invariant.

This dynamic behavior of degrees is consistent with the static measure of the degree distribution. Using the density conservation law of degree distribution over evolution
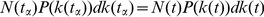
(18)the degree distribution Equation (2), and the growth laws Equations (14) and (17), the following relationship between the static exponent 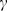 and the dynamic rates 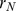 and 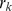 was obtained:
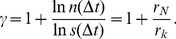
(19)


Equation (19) was tested in Figures S7D and S8D for scale-free networks (such as *M. musculus*, *C. elegans*, *D. melanogaster* and *E. coli*). For exponential networks (such as *S. cerevisiae*, *H. sapiens* and *A. thaliana*), Equation (19) suggests 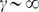 since 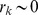 as measured in Figure 4E and Figure S6E. The comparison between 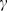 and 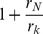 is shown in Table 4 with good agreements.

The relationship between 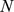, 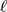 and 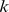 is closed by the third equation:
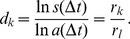
(20)


This was tested in Figures S7E and S8E for scale-free networks. For exponential networks, we found 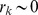, and therefore 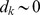, which agrees with the static measurement (Table 4).

Equations (16), (19), and (20) relate the static exponents 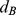, 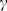, and 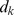 to the dynamic growth rates 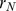, 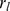, and 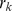. Combining the three equations together, the static relationship Equation (5) is recovered, which is originally derived from scaling argument [28].

Similar to the growth laws of 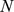, 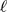 and 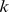, an exponential growth of 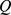 is assumed:
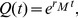
(21) and a relationship is predicted as:
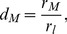
(22)


This assumes that the modularity exponent 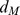 is invariant during evolution. Direct test of this assumption would require detailed analysis of network structure and protein functions, which was left for future study.

The above results are summarized in Figure 5. At the core of the results is the exponential growth of the system quantities (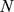, 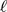, 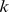, 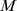), and the relations between the static exponents (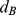, 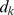, 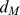, 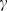) and dynamic rates (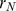, 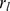, 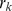, 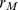). Therefore, the multiplicative growth provides a fundamental mechanism for the evolutionary principle of PPI networks.

## Supporting Information

Figure S1
**Distribution of STRING experimental scores.** Box-and-whisker plots showing the distribution of STRING experimental scores for the organisms investigated. The filter threshold 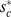 for each species is indicated by a red line. The plots were created using the boxplot function of R.(TIF)

Figure S2
**Determine the present-day PPI networks.** (A) Percolation analysis of the present-day *S. cerevisiae* and *M. musculus* PPI networks from the STRING database. We plot the size of the largest (black) and second largest (red, rescaled and shifted) connected components (as measured by the fraction to the total number of nodes) versus cutoff score 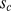. The first jump of the largest connected component corresponds to the threshold 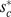. Inset shows schematically an uncorrelated percolation. (B) Demonstration of the box-covering algorithm MEMB [28], [60] for a schematic network. The network is covered with boxes of size 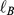. (C) Plot of the number of boxes 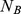 versus box size 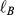 at different 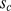. (D) 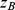 versus 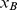 under renormalization at different 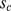. The dashed line indicates the small-world to fractal transition point 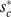.(TIF)

Figure S3
**Modularity of PPI networks.** Log-log plot of the modularity ratio 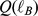 versus size of the modules 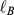. Each point is an average over many modules with the same binned 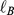. The error bars are the standard deviations.(TIF)

Figure S4 **Overlap of the different networks used for the study.** The overlaps between the networks of all organisms on all evolutionary levels are shown, with the number of overlapping nodes in (A) and the number of overlapping interactions in (B). The color intensities represent the relative abundances in a heat map-like manner, whith the lightest/darkest color referring to the lowest/highest number in the whole table except the diagonal. For example, while the interactome sizes are similar in *M. musculus* and *A. thaliana*, the large overlap between the interactomes of *H. sapiens* and *M. musculus* can be attributed to their closer evolutionary relationship. In case of equal evolutionary distances, the size of the interactome is decisive for the overlap; e.g. the overlap between *E. coli* and *S. cerevisiae* is larger than the one between *E. coli* and *C. elegans*. In many cases, the overlaps in the ancient networks get smaller, which reflects the smaller network sizes.
(TIF)

Figure S5
**Degree distribution** 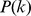 **of PPI networks.** Left, semi-log plot of 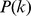 shows that the degree distribution of the *S. cerevisiae* PPI network is exponential. Right, log-log plot of 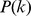 shows that the degree distribution of the *M. musculus* PPI network is scale-free (power-law) with degree exponent 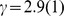.(TIF)

Figure S6
**Multiplicative growth mechanism of the** ***H. sapiens*** **PPI network.** (A) 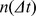 vs. 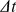. (B) Scaling between 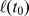 and 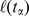. (C) 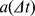 vs. 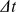. (D) 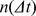 vs. a(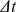). (E) Scaling between 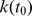 and 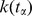. This figure is analogous to Figure 4 for *S. cerevisiae*.(TIF)

Figure S7
**Multiplicative growth mechanism of the** ***M. musculus*** **PPI network.** (A) Scaling between 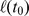 and 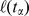. (B) 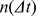 vs. 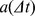. (C)Scaling between 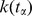 and 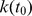. (D) 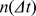 vs. 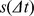. (E) 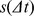 vs. 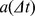. This figure is analogous to Figure 4 for *S. cerevisiae*. Different from *S. cerevisiae*, which has an exponential degree distribution, *M. musculus* has a power-law (scale-free) degree distribution (see Figure S5).(TIF)

Figure S8
**Multiplicative growth mechanism of the** ***E. coli*** **PPI network.** (A) Scaling between 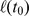 and 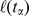. (B) 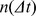 vs. 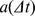. (C) Scaling between 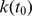 and 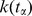. (D) 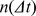 vs. 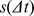. (E) 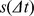 vs. 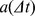. This figure is analogous to Figure 4 for *S. cerevisiae*.(TIF)

Figure S9
**Duplication-divergence model.** (A) The two basic modes for the model. Left, mode I: protein A and B interact to each other before duplication, and protein A duplicates to A and A′. After duplication, A and A′ have equal probability 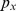 to keep the interaction with B. Right, mode II: protein A and B do not interact before duplication. After duplication, A and A′ have equal probability 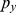 to generate a new interaction with B. (B) Protein A and B duplicate to two clusters of 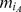 and 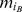 proteins respectively after 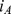 and 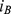 duplications. We have 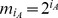, 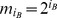, the total number of duplications 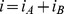, and the total number of possible links between cluster A and B 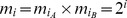. 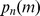 is the probability to have 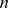 interactions out of the 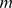 total possible ones. (C) An example of distance growth in the duplication-divergence model. Left, distance 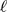 between two proteins (red circles) does not change when 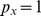 (pure duplication of green circles, without divergence). Right, 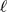 increases when 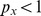 due to the loss of interactions. The red nodes are connected through a long path of interactions between existing proteins (blue circles).(TIF)

Figure S10
**The scaling of** 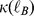 **vs.** 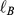 **.** The renormalized degree exponent 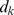 is calculated according to Equation (4). As an example, the inset shows the renormalization relation 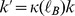 for the case 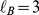.(TIF)

Figure S11
**Fitting parameters and testing the duplication-divergence model.** Fit of 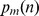 to the empirical data of (A) *S. cerevisiae*, (B) *M. musculus*, and (C) *H. sapiens*. The curves are the fitted theoretical values, and the scatters are the empirical data. The model and the data are in good agreement. Parameters 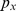, 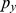 and 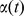 (one 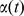 for each time level 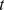) of each species are determined from this fitting.(TIF)

Table S1
**Node and interaction counts at each filter step.** Numbers of proteins and interactions at each filter step preceding the network construction and analysis. Four different filters were applied: STRING experimental score 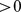, conservation on all evolutionary levels defined for the corresponding organism in eggNOG, filtering at the percolation threshold 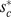, and filtering at the percolation threshold 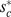 and considering only the largest connected component. The largest component (which is also called giant component in the percolation literatures [62]) is required for the topological analysis.(PDF)

Table S2 **Divergence times.** Estimated divergence times for the evolutionary levels in the eggNOG database. They represent the time point when the last common ancestor of a certain evolutionary level existed. Estimates are derived from the TimeTree database [74].
(PDF)

## References

[1] Mika S, Rost B (2006) Protein-protein interactions more conserved within species than across species. PLoS Comput Biol 2: e79. 16854211 10.1371/journal.pcbi.0020079 PMC1513270

[2] Zinman GE, Zhong S, Bar-Joseph Z (2011) Biological interaction networks are conserved at the module level. BMC Syst Biol 5: 134. 21861884 10.1186/1752-0509-5-134 PMC3212960

[3] Gibson TA, Goldberg DS (2011) Improving evolutionary models of protein interaction networks. Bioinformatics 27: 376–382. 21067999 10.1093/bioinformatics/btq623 PMC3031028

[4] Fields S (2005) High-throughput two-hybrid analysis. FEBS J 272: 5391–5399. 16262681 10.1111/j.1742-4658.2005.04973.x

[5] Suter B, Kittanakom S, Stagljar I (2008) Two-hybrid technologies in proteomics research. Curr Opin Biotechnol 19: 316–323. 18619540 10.1016/j.copbio.2008.06.005

[6] Koegl M, Uetz P (2007) Improving yeast two-hybrid screening systems. Brief Funct Genomic Proteomic 6: 302–312. 18218650 10.1093/bfgp/elm035

[7] Gavin AC, Aloy P, Grandi P, Krause R, Boesche M, et al (2006) Proteome survey reveals modularity of the yeast cell machinery. Nature 440: 631–636. 16429126 10.1038/nature04532

[8] Krogan NJ, Cagney G, Yu H, Zhong G, Guo X, et al (2006) Global landscape of protein complexes in the yeast saccharomyces cerevisiae. Nature 440: 637–643. 16554755 10.1038/nature04670

[9] Wodak SJ, Pu S, Vlasblom J, Séraphin B (2009) Challenges and rewards of interaction proteomics. Mol Cell Proteomics 8: 3–18. 18799807 10.1074/mcp.R800014-MCP200

[10] Tarassov K, Messier V, Landry CR, Radinovic S, Serna Molina MM, et al (2008) An in vivo map of the yeast protein interactome. Science 320: 1465–1470. 18467557 10.1126/science.1153878

[11] Yu H, Braun P, Yıldırım MA, Lemmens I, Venkatesan K, et al (2008) High-quality binary protein interaction map of the yeast interactome network. Science 322: 104–110. 18719252 10.1126/science.1158684 PMC2746753

[12] von Mering C, Krause R, Snel B, Cornell M, Oliver SG, et al (2002) Comparative assessment of large-scale data sets of protein-protein interactions. Nature 417: 399–403. 12000970 10.1038/nature750

[13] Huang H, Jedynak BM, Bader JS (2007) Where have all the interactions gone? estimating the coverage of two-hybrid protein interaction maps. PLoS Comput Biol 3: e214. 18039026 10.1371/journal.pcbi.0030214 PMC2082503

[14] Braun P, Tasan M, Dreze M, Barrios-Rodiles M, Lemmens I, et al (2009) An experimentally derived confidence score for binary protein-protein interactions. Nat Methods 6: 91–97. 19060903 10.1038/nmeth.1281 PMC2976677

[15] Rajagopala SV, Hughes KT, Uetz P (2009) Benchmarking yeast two-hybrid systems using the interactions of bacterial motility proteins. Proteomics 9: 5296–5302. 19834901 10.1002/pmic.200900282 PMC2818629

[16] Collins SR, Kemmeren P, Zhao XC, Greenblatt JF, Spencer F, et al (2007) Toward a comprehensive atlas of the physical interactome of saccharomyces cerevisiae. Mol Cell Proteomics 6: 439–450. 17200106 10.1074/mcp.M600381-MCP200

[17] Rual J, Venkatesan K, Hao T, Hirozane-Kishikawa T, Dricot A, et al (2005) Towards a proteomescale map of the human protein-protein interaction network. Nature 437: 1173–1178. 16189514 10.1038/nature04209

[18] Arabidopsis Interactome Mapping Consortium (2011) Evidence for network evolution in an Arabidopsis interactome map. Science 333: 601–607. 21798944 10.1126/science.1203877 PMC3170756

[19] Licata L, Briganti L, Peluso D, Perfetto L, Iannuccelli M, et al (2012) MINT, the molecular interaction database: 2012 update. Nucleic Acids Res 40: D857–861. 22096227 10.1093/nar/gkr930 PMC3244991

[20] Salwinski L, Miller CS, Smith AJ, Pettit FK, Bowie JU, et al (2004) The database of interacting proteins: 2004 update. Nucleic Acids Res 32: D449–451. 14681454 10.1093/nar/gkh086 PMC308820

[21] Breitkreutz B, Stark C, Reguly T, Boucher L, Breitkreutz A, et al (2008) The BioGRID interaction database: 2008 update. Nucleic Acids Res 36: D637–640. 18000002 10.1093/nar/gkm1001 PMC2238873

[22] Kerrien S, Aranda B, Breuza L, Bridge A, Broackes-Carter F, et al (2012) The IntAct molecular interaction database in 2012. Nucleic Acids Res 40: D841–846. 22121220 10.1093/nar/gkr1088 PMC3245075

[23] Szklarczyk D, Franceschini A, Kuhn M, Simonovic M, Roth A, et al (2011) The STRING database in 2011: functional interaction networks of proteins, globally integrated and scored. Nucleic Acids Res 39: D561–568. 21045058 10.1093/nar/gkq973 PMC3013807

[24] Barabasi AL, Oltvai ZN (2004) Network biology: understanding the cell's functional organization. Nat Rev Genet 5: 101–113. 14735121 10.1038/nrg1272

[25] Bollobás B (1985) Random graphs. London: Academic Press.

[26] Watts DJ, Strogatz SH (1998) Collective dynamics of ‘small-world’ networks. Nature 393: 440–442. 9623998 10.1038/30918

[27] Ravasz E, Somera AL, Mongru DA, Oltvai ZN, Barabási AL (2002) Hierarchical organization of modularity in metabolic networks. Science 297: 1551–1555. 12202830 10.1126/science.1073374

[28] Song C, Havlin S, Makse HA (2005) Self-similarity of complex networks. Nature 433: 392–395. 15674285 10.1038/nature03248

[29] Albert R, Jeong H, Barabási AL (2000) Error and attack tolerance of complex networks. Nature 406: 378–382. 10935628 10.1038/35019019

[30] Albert R, Barabási AL (2002) Statistical mechanics of complex networks. Rev Mod Phys 74: 47–97.

[31] Barabási AL, Albert R (1999) Emergence of scaling in random networks. Science 286: 509–512. 10521342 10.1126/science.286.5439.509

[32] Wagner A (2001) The yeast protein interaction network evolves rapidly and contains few redundant duplicate genes. Mol Biol Evol 18: 1283–1292. 11420367 10.1093/oxfordjournals.molbev.a003913

[33] Song C, Havlin S, Makse HA (2006) Origins of fractality in the growth of complex networks. Nat Physics 2: 275–281.

[34] Gallos LK, Song C, Havlin S, Makse HA (2007) Scaling theory of transport in complex biological networks. Proc Natl Acad Sci 104: 7746–7751. 17470793 10.1073/pnas.0700250104 PMC1876518

[35] Galvao V, Miranda JGV, Andrade RFS, Andrade JS, Gallos LK, et al (2010) Modularity map of the network of human cell differentiation. Proc Natl Acad Sci 107: 5750–5755. 20220102 10.1073/pnas.0914748107 PMC2851936

[36] Goh KI, Salvi G, Kahng B, Kim D (2006) Skeleton and fractal scaling in complex networks. Phys Rev Lett 96: 018701. 16486532 10.1103/PhysRevLett.96.018701

[37] Presser A, Elowitz MB, Kellis M, Kishony R (2008) The evolutionary dynamics of the saccharomyces cerevisiae protein interaction network after duplication. Proc Natl Acad Sci 105: 950–954. 18199840 10.1073/pnas.0707293105 PMC2242688

[38] Ohno S (1970) Evolution by gene duplication. Berlin:Springer-Verlag.

[39] Li WS (1997) Molecular evolution. Sunderland,MA:Sinauer Associates, Inc.

[40] Patthy L (1999) Protein evolution. Portland, OR:Blackwell Publishers.

[41] Taylor JS, Raes J (2004) Duplication and divergence: the evolution of new genes and old ideas. Annu Rev Genet 38: 615–643. 15568988 10.1146/annurev.genet.38.072902.092831

[42] Wagner A (2003) How the global structure of protein interaction networks evolves. Proc Biol Sc 270: 457–466. 12641899 10.1098/rspb.2002.2269 PMC1691265

[43] Qian W, He X, Chan E, Xu H, Zhang J (2011) Measuring the evolutionary rate of protein-protein interaction. Proc Natl Acad Sci 108: 8725–8730. 21555556 10.1073/pnas.1104695108 PMC3102417

[44] Evlampiev K, Isambert H (2007) Modeling protein network evolution under genome duplication and domain shuffling. BMC Syst Biol 1. 10.1186/1752-0509-1-49 PMC2245809 17999763

[45] Evlampiev K, Isambert H (2008) Conservation and topology of protein interaction networks under duplication-divergence evolution. Proc Natl Acad Sci 105: 9863–9868. 18632555 10.1073/pnas.0804119105 PMC2481380

[46] Sole RV, Pastor-Satorras R, Smith E, Kepler TB (2002) A model of large-scale proteome evolution. Adv Complex Syst 5: 43.

[47] Kim J, Krapivsky PL, Kahng B, Redner S (2002) Infinite-order percolation and giant fluctuations in a protein interaction network. Phys Rev E 66: 055101. 10.1103/PhysRevE.66.055101 12513542

[48] Chung F, Lu L, Dewey TG, Galas DJ (2002) Duplication models for biological networks. J Comput Biol 10: 677. 10.1089/106652703322539024 14633392

[49] Vazquez A, Flammini A, Maritan A, Vespignani A (2003) Modeling of protein interaction networks. Complexus 1: 38. 10.1038/nbt825 12740586

[50] Mirkin BG, Fenner TI, Galperin MY, Koonin EV (2003) Algorithms for computing parsimonious evolutionary scenarios for genome evolution, the last universal common ancestor and dominance of horizontal gene transfer in the evolution of prokaryotes. BMC evolutionary biology 3: 2. 12515582 10.1186/1471-2148-3-2 PMC149225

[51] Patro R, Sefer E, Malin J, Marais G, Navlakha S, et al (2012) Parsimonious reconstruction of network evolution. Algorithms for molecular biology: AMB 7: 25. 22992218 10.1186/1748-7188-7-25 PMC3492119

[52] Navlakha S, Kingsford C (2011) Network archaeology: Uncovering ancient networks from presentday interactions. PLoS Comput Biol 7: e1001119. 21533211 10.1371/journal.pcbi.1001119 PMC3077358

[53] Zhang X, Moret BME (2010) Refining transcriptional regulatory networks using network evolutionary models and gene histories. Algorithms for molecular biology: AMB 5: 1. 20047657 10.1186/1748-7188-5-1 PMC2823753

[54] Pinney JW, Amoutzias GD, Rattray M, Robertson DL (2007) Reconstruction of ancestral protein interaction networks for the bZIP transcription factors. Proceedings of the National Academy of Sciences of the United States of America 104: 20449–20453. 18077348 10.1073/pnas.0706339104 PMC2154451

[55] Gibson TA, Goldberg DS (2009) Reverse engineering the evolution of protein interaction networks. Pacific Symposium on Biocomputing Pacific Symposium on Biocomputing: 190–202. 19213136

[56] Dutkowski J, Tiuryn J (2007) Identification of functional modules from conserved ancestral protein protein interactions. Bioinformatics 23: i149–158. 17646291 10.1093/bioinformatics/btm194

[57] Koonin EV (2005) Orthologs, paralogs, and evolutionary genomics. Annu Rev Genet 39: 309–338. 16285863 10.1146/annurev.genet.39.073003.114725

[58] Kunin V, Pereira-Leal JB, Ouzounis CA (2004) Functional evolution of the yeast protein interaction network. Mol Biol Evo 21: 1171–1176. 10.1093/molbev/msh085 15071090

[59] Muller J, Szklarczyk D, Julien P, Letunic I, Roth A, et al (2010) eggNOG v2.0: extending the evolutionary genealogy of genes with enhanced non-supervised orthologous groups, species and functional annotations. Nucleic Acids Res 38: D190–195. 19900971 10.1093/nar/gkp951 PMC2808932

[60] Song C, Gallos LK, Havlin S, Makse HA (2007) How to calculate the fractal dimension of a complex network: the box covering algorithm. J Stat Mech: Theory Exp 3: P03006.

[61] Rozenfeld HD, Song C, Makse HA (2010) Small-world to fractal transition in complex networks: A renormalization group approach. Phys Rev Lett 104: 025701. 20366610 10.1103/PhysRevLett.104.025701

[62] Bunde A, Havlin S, editors (1996) Fractals and disordered systems, 2nd edition. New York: Springer-Verlag.

[63] Jeong H, Mason SP, Barabási AL, Oltvai ZN (2001) Lethality and centrality in protein networks. Nature 411: 41–42. 11333967 10.1038/35075138

[64] Amaral LAN, Scala A, Barthélémy M, Stanley HE (2000) Classes of small-world networks. Proc Natl Acad Sci 971: 11149–11152. 10.1073/pnas.200327197 PMC17168 11005838

[65] Jensen LJ, Julien P, Kuhn M, von Mering C, Muller J, et al (2008) eggNOG: automated construction and annotation of orthologous groups of genes. Nucleic Acids Res 36: D250–254. 17942413 10.1093/nar/gkm796 PMC2238944

[66] Gallos LK, Makse HA, Sigman M (2012) A small world of weak ties provides optimal global integration of self-similar modules in functional brain networks. Proc Natl Acad Sci 109: 2825–2830. 22308319 10.1073/pnas.1106612109 PMC3286928

[67] Granovetter MS (1973) The strength of weak ties. Am J Sociol 78 pp. 1360–1380.

[68] Gibson TA, Goldberg DS (2009) Questioning the ubiquity of neofunctionalization. PLoS Comput Biol 5: e1000252. 19119408 10.1371/journal.pcbi.1000252 PMC2597716

[69] Yang L, Pei W, Li T, Cao Y, Shen Y, et al.. (2008) A fractal network model with tunable fractal dimension. In: Neural Networks and Signal Processing, 2008 International Conference on. pp.53–57.

[70] Bizhani G, Sood V, Paczuski M, Grassberger P (2011) Random sequential renormalization of networks: Application to critical trees. Phys Rev E 83: 036110. 10.1103/PhysRevE.83.036110 21517561

[71] Sayers EW, Barrett T, Benson DA, Bolton E, Bryant SH, et al (2012) Database resources of the national center for biotechnology information. Nucleic Acids Res 40: D13–25. 22140104 10.1093/nar/gkr1184 PMC3245031

[72] Gallos LK, Barttfeld P, Havlin S, Sigman M, Makse HA (2012) Collective behavior in the spatial spreading of obesity. Sci Rep 2: 454. 22822425 10.1038/srep00454 PMC3400682

[73] Girvan M, Newman MEJ (2002) Community structure in social and biological networks. Proc Natl Acad Sci 99: 7821–7826. 12060727 10.1073/pnas.122653799 PMC122977

[74] Hedges SB, Dudley J, Kumar S (2006) TimeTree: a public knowledge-base of divergence times among organisms. Bioinformatics 22: 2971–2972. 17021158 10.1093/bioinformatics/btl505

