# Supplementary figures and images for: The Evolutionary Dynamics of Protein-Protein Interaction Networks Inferred from the Reconstruction of Ancient Networks
Source: PLoS One. 2013 Mar 20;8(3):e58134. doi: 10.1371/journal.pone.0058134 (PMC3603955; doi:10.1371/journal.pone.0058134)

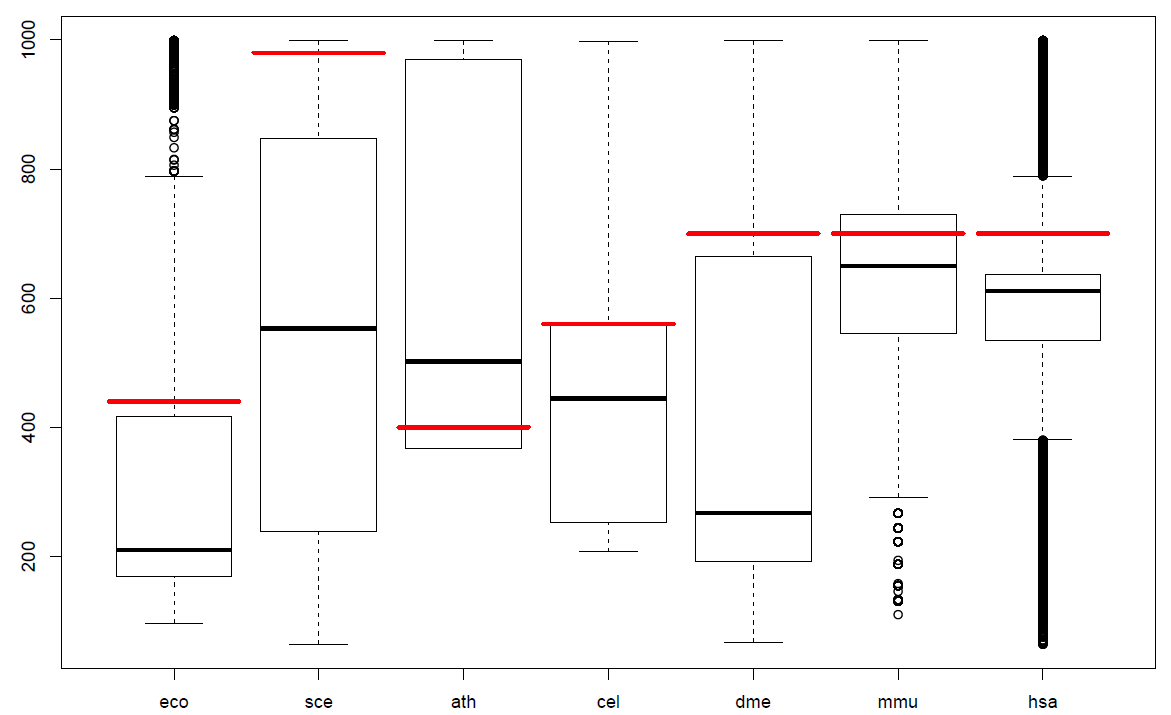

Supplement: Figure S1 — Distribution of STRING experimental scores. Box-and-whisker plots showing the distribution of STRING experimental scores for the organisms investigated. The filter threshold for each species is indicated by a red line. The plots were created using the boxplot function of R. (TIF) [file pone.0058134.s001.tif]

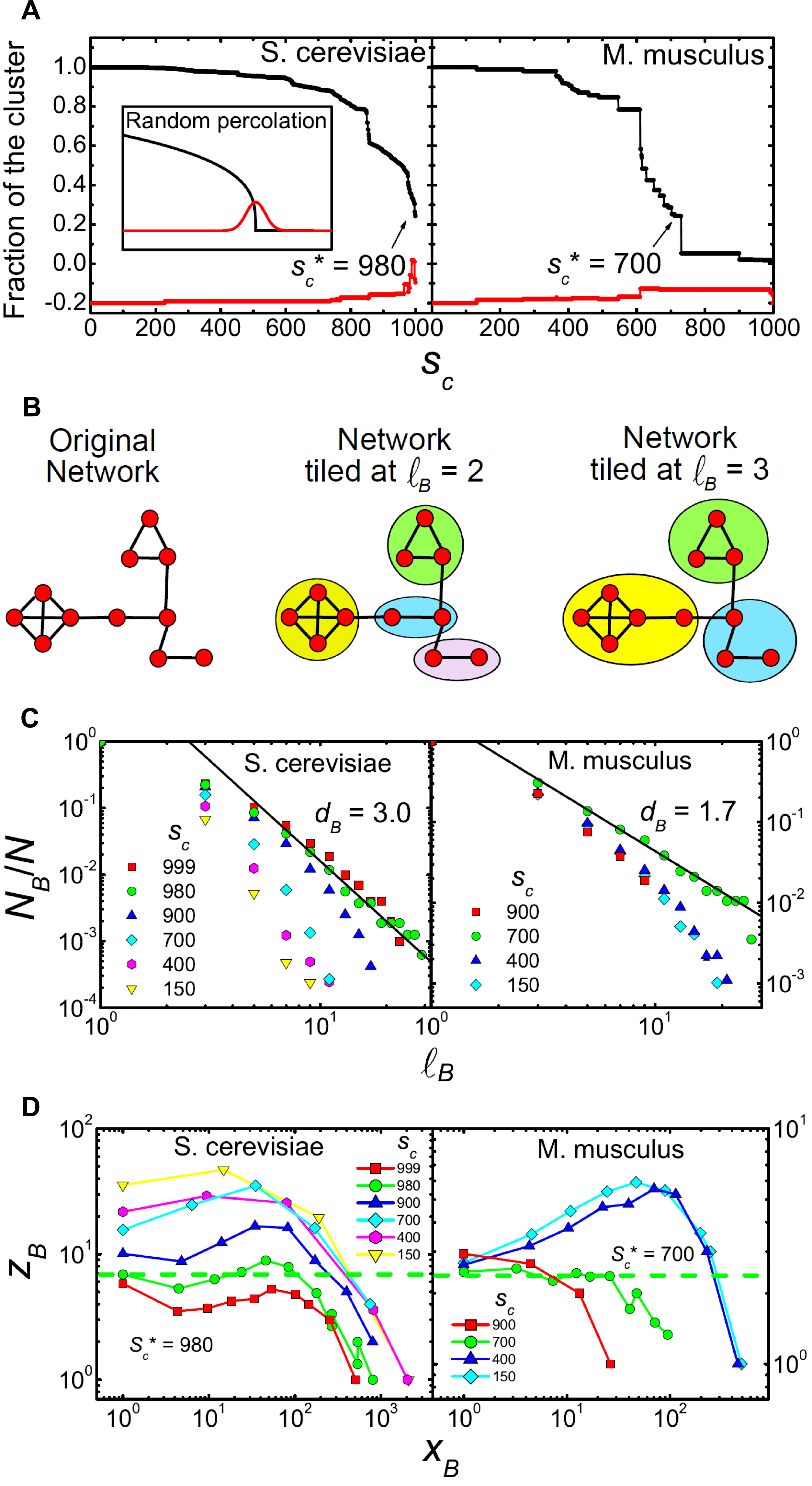

Supplement: Figure S2 — Determine the present-day PPI networks. (A) Percolation analysis of the present-day S. cerevisiae and M. musculus PPI networks from the STRING database. We plot the size of the largest (black) and second largest (red, rescaled and shifted) connected components (as measured by the fraction to the total number of nodes) versus cutoff score . The first jump of the largest connected component corresponds to the threshold . Inset shows schematically an uncorrelated percolation. (B) Demonstration of the box-covering algorithm MEMB [28], [60] for a schematic network. The network is covered with boxes of size . (C) Plot of the number of boxes versus box size at different . (D) versus under renormalization at different . The dashed line indicates the small-world to fractal transition point . (TIF) [file pone.0058134.s002.tif]

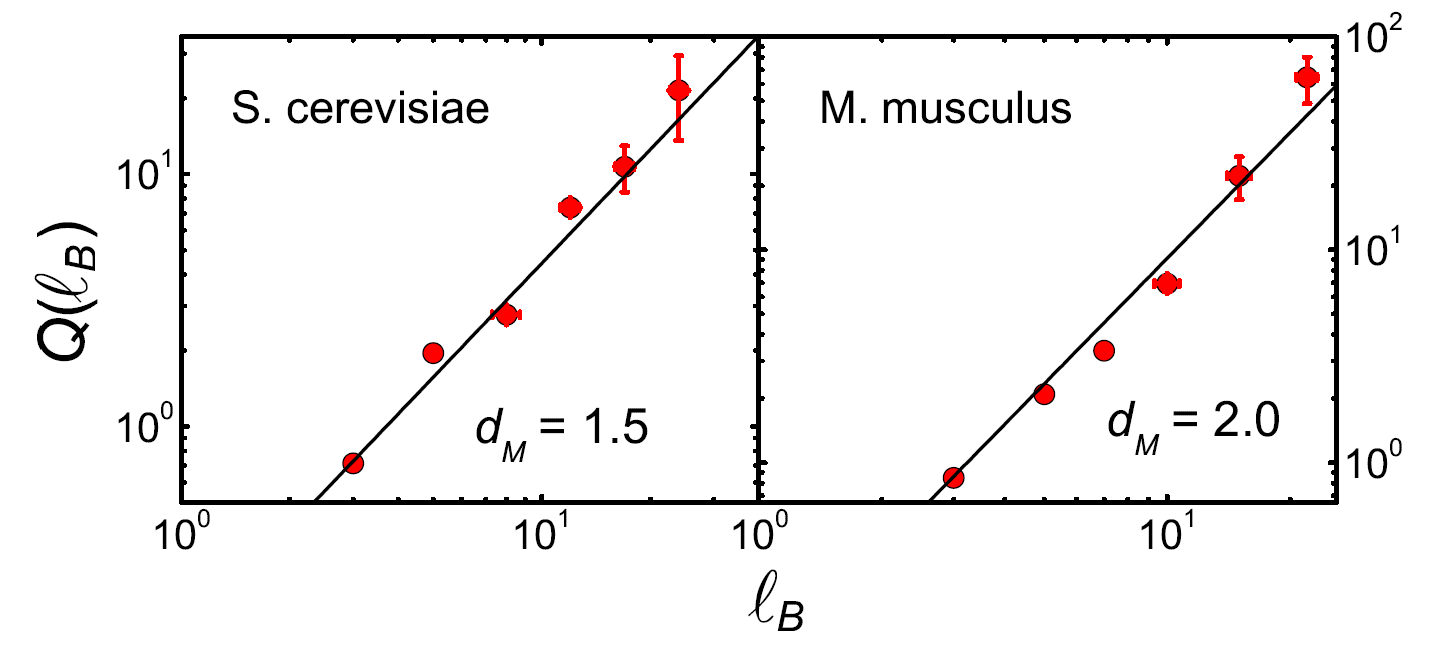

Supplement: Figure S3 — Modularity of PPI networks. Log-log plot of the modularity ratio versus size of the modules . Each point is an average over many modules with the same binned . The error bars are the standard deviations. (TIF) [file pone.0058134.s003.tif]

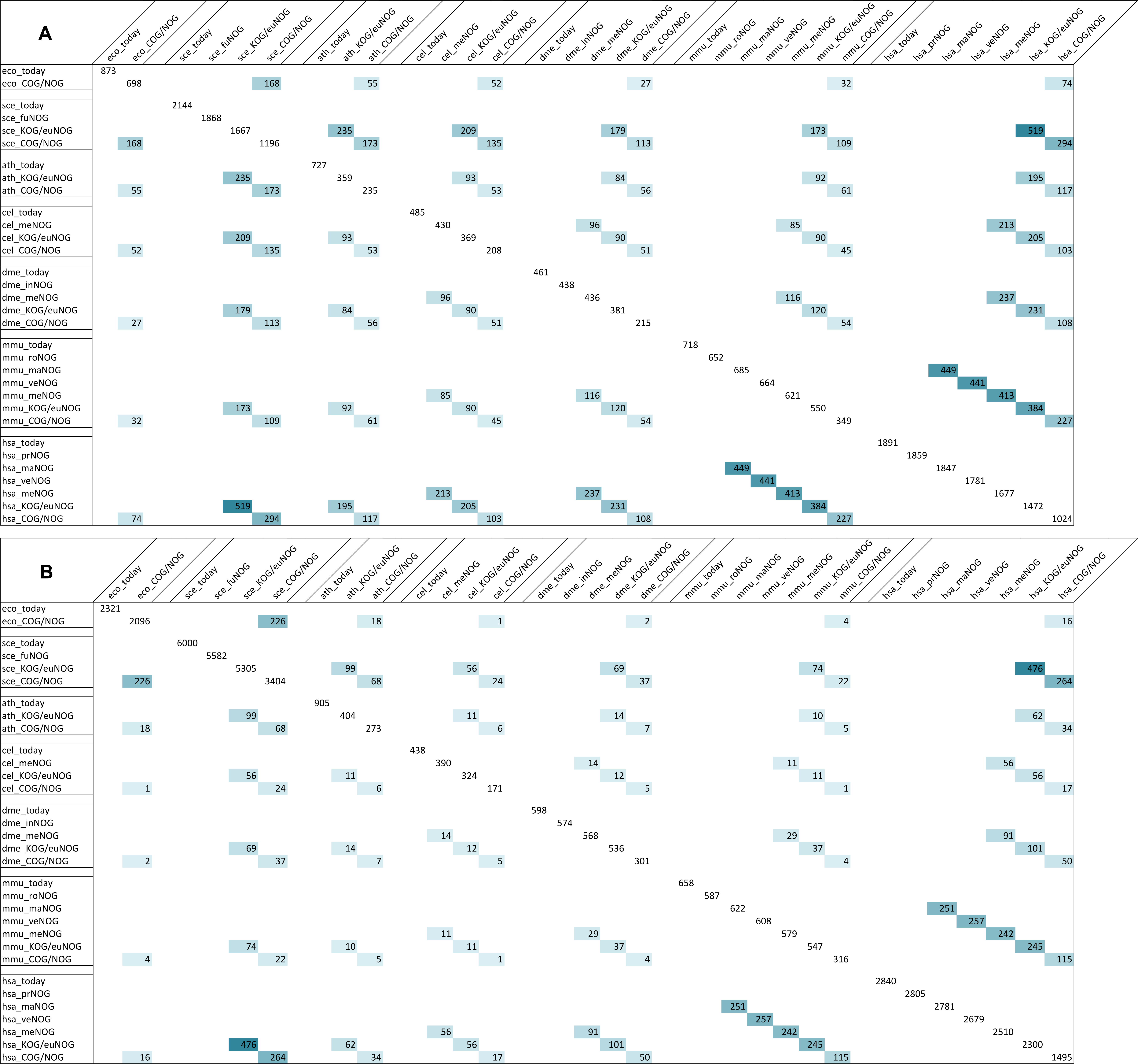

Supplement: Figure S4 — Overlap of the different networks used for the study. The overlaps between the networks of all organisms on all evolutionary levels are shown, with the number of overlapping nodes in (A) and the number of overlapping interactions in (B). The color intensities represent the relative abundances in a heat map-like manner, whith the lightest/darkest color referring to the lowest/highest number in the whole table except the diagonal. For example, while the interactome sizes are similar in M. musculus and A. thaliana, the large overlap between the interactomes of H. sapiens and M. musculus can be attributed to their closer evolutionary relationship. In case of equal evolutionary distances, the size of the interactome is decisive for the overlap; e.g. the overlap between E. coli and S. cerevisiae is larger than the one between E. coli and C. elegans. In many cases, the overlaps in the ancient networks get smaller, which reflects the smaller network sizes. (TIF) [file pone.0058134.s004.tif]

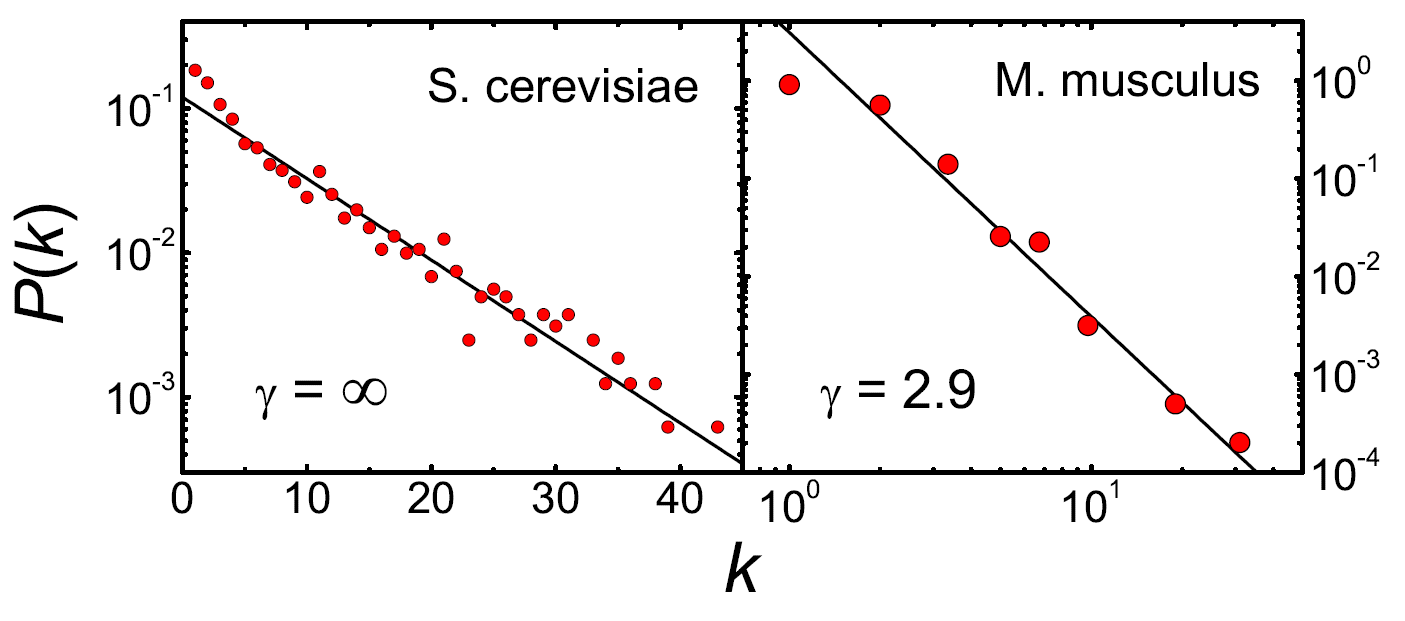

Supplement: Figure S5 — Degree distribution of PPI networks. Left, semi-log plot of shows that the degree distribution of the S. cerevisiae PPI network is exponential. Right, log-log plot of shows that the degree distribution of the M. musculus PPI network is scale-free (power-law) with degree exponent . (TIF) [file pone.0058134.s005.tif]

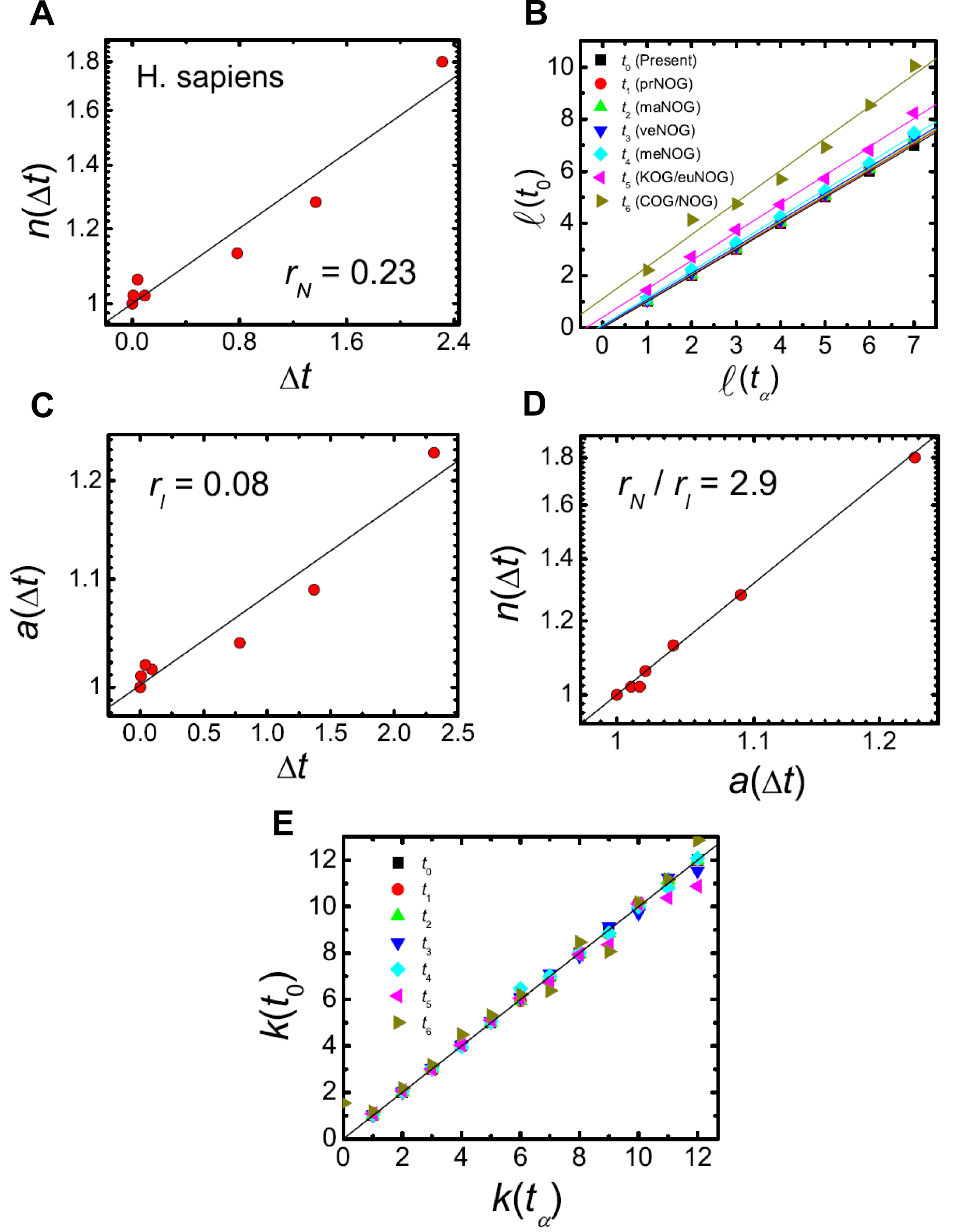

Supplement: Figure S6 — Multiplicative growth mechanism of the H. sapiens PPI network. (A) vs. . (B) Scaling between and . (C) vs. . (D) vs. a(). (E) Scaling between and . This figure is analogous to Figure 4 for S. cerevisiae. (TIF) [file pone.0058134.s006.tif]

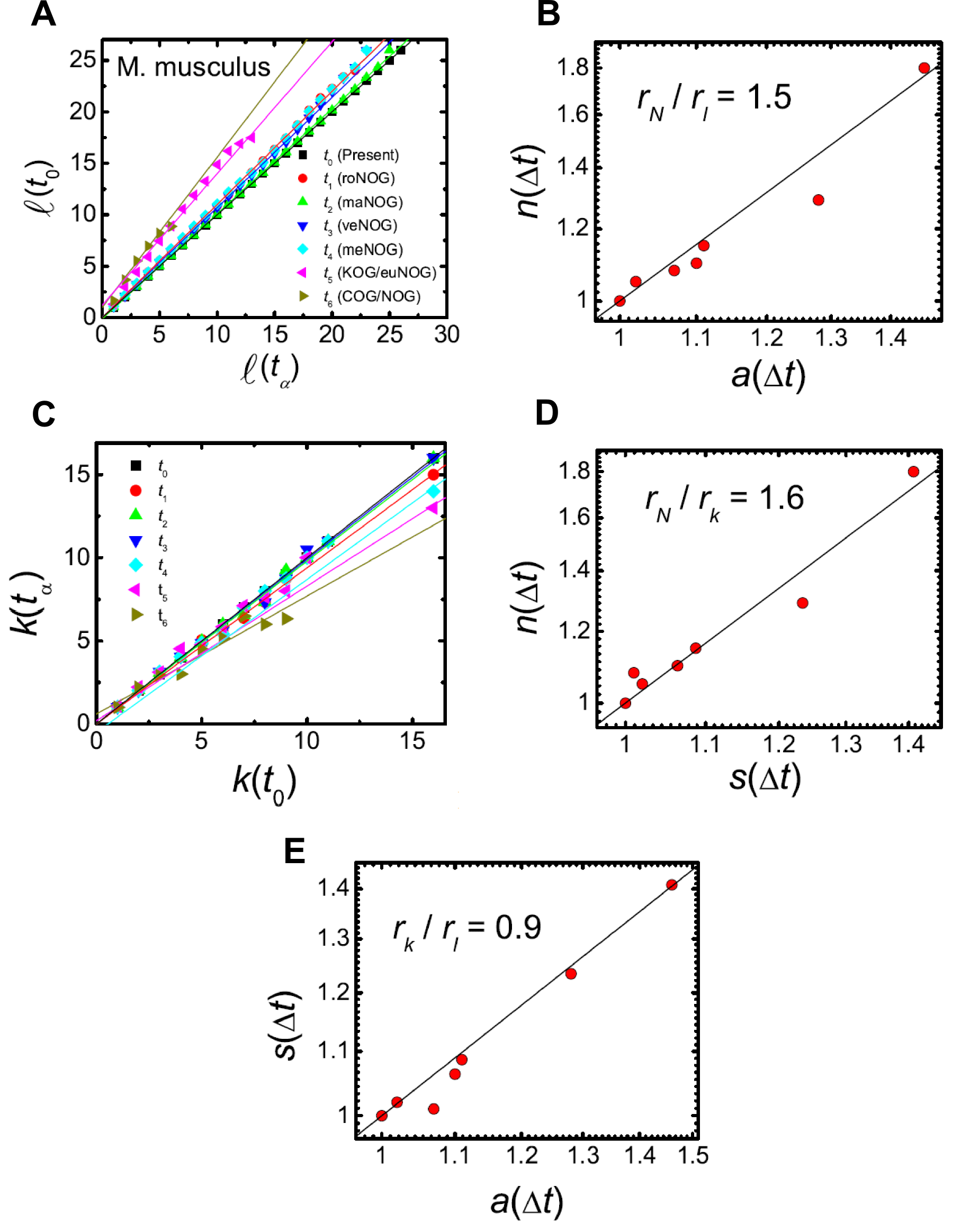

Supplement: Figure S7 — Multiplicative growth mechanism of the M. musculus PPI network. (A) Scaling between and . (B) vs. . (C)Scaling between and . (D) vs. . (E) vs. . This figure is analogous to Figure 4 for S. cerevisiae. Different from S. cerevisiae, which has an exponential degree distribution, M. musculus has a power-law (scale-free) degree distribution (see Figure S5). (TIF) [file pone.0058134.s007.tif]

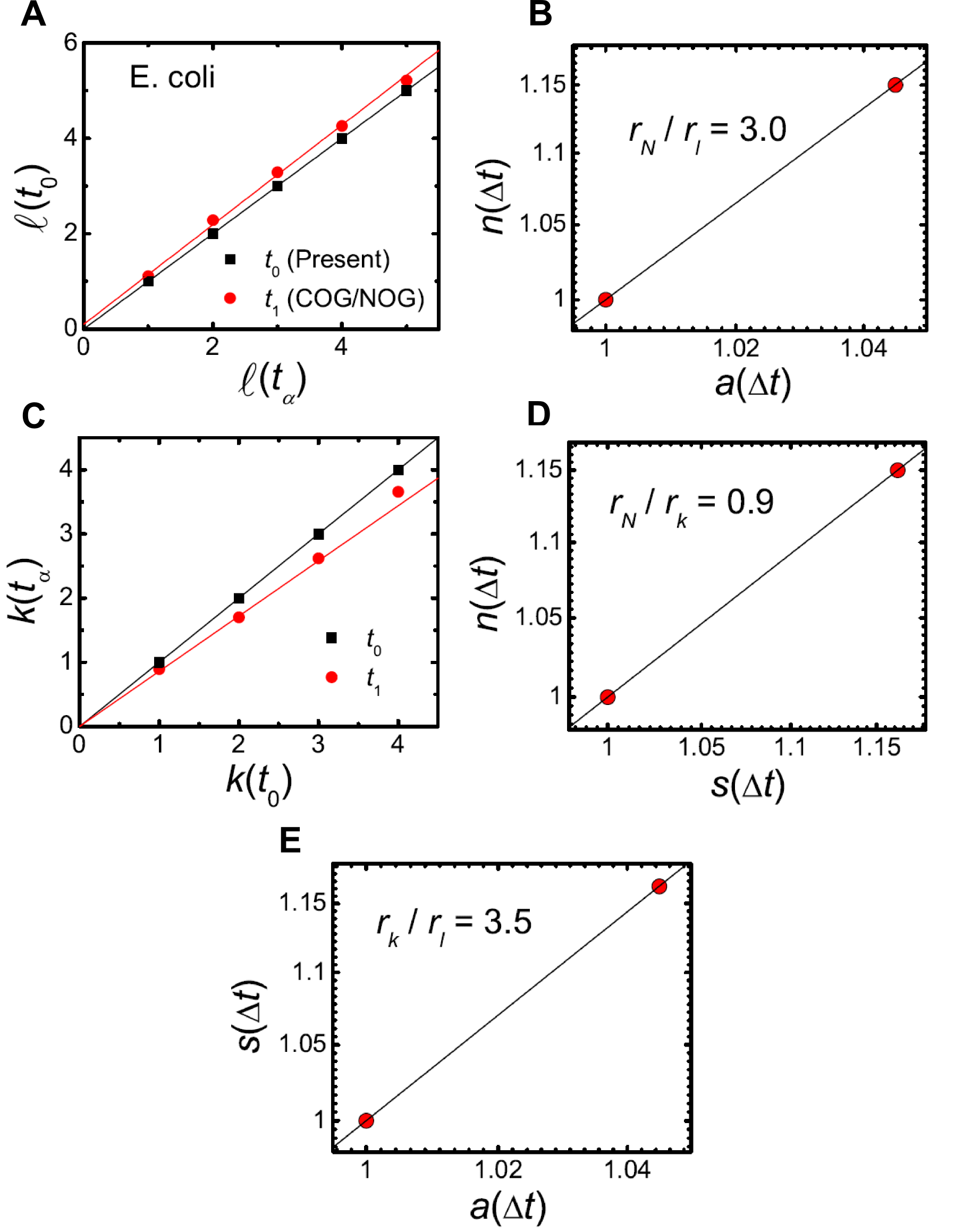

Supplement: Figure S8 — Multiplicative growth mechanism of the E. coli PPI network. (A) Scaling between and . (B) vs. . (C) Scaling between and . (D) vs. . (E) vs. . This figure is analogous to Figure 4 for S. cerevisiae. (TIF) [file pone.0058134.s008.tif]

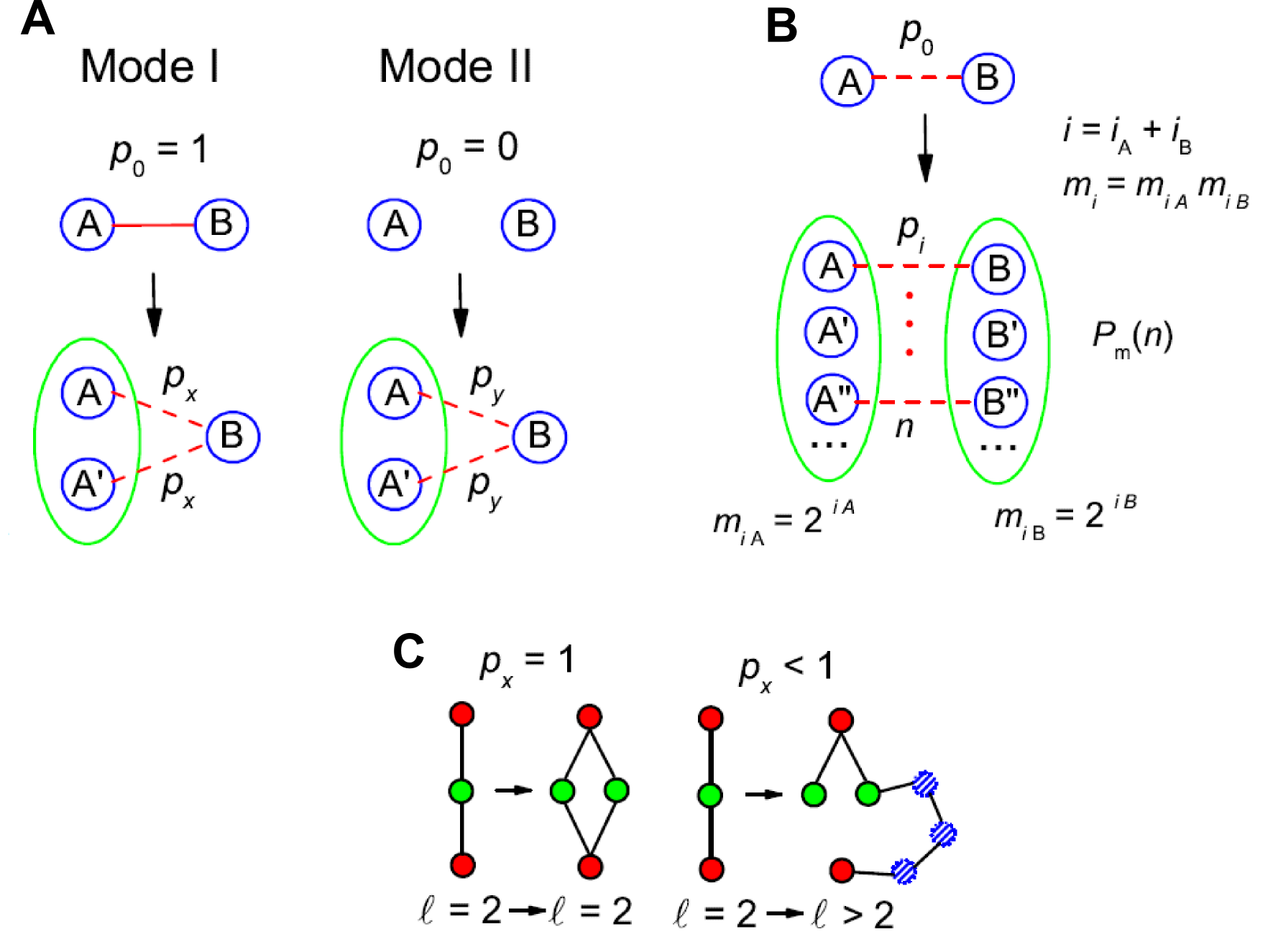

Supplement: Figure S9 — Duplication-divergence model. (A) The two basic modes for the model. Left, mode I: protein A and B interact to each other before duplication, and protein A duplicates to A and A′. After duplication, A and A′ have equal probability to keep the interaction with B. Right, mode II: protein A and B do not interact before duplication. After duplication, A and A′ have equal probability to generate a new interaction with B. (B) Protein A and B duplicate to two clusters of and proteins respectively after and duplications. We have , , the total number of duplications , and the total number of possible links between cluster A and B . is the probability to have interactions out of the total possible ones. (C) An example of distance growth in the duplication-divergence model. Left, distance between two proteins (red circles) does not change when (pure duplication of green circles, without divergence). Right, increases when due to the loss of interactions. The red nodes are connected through a long path of interactions between existing proteins (blue circles). (TIF) [file pone.0058134.s009.tif]

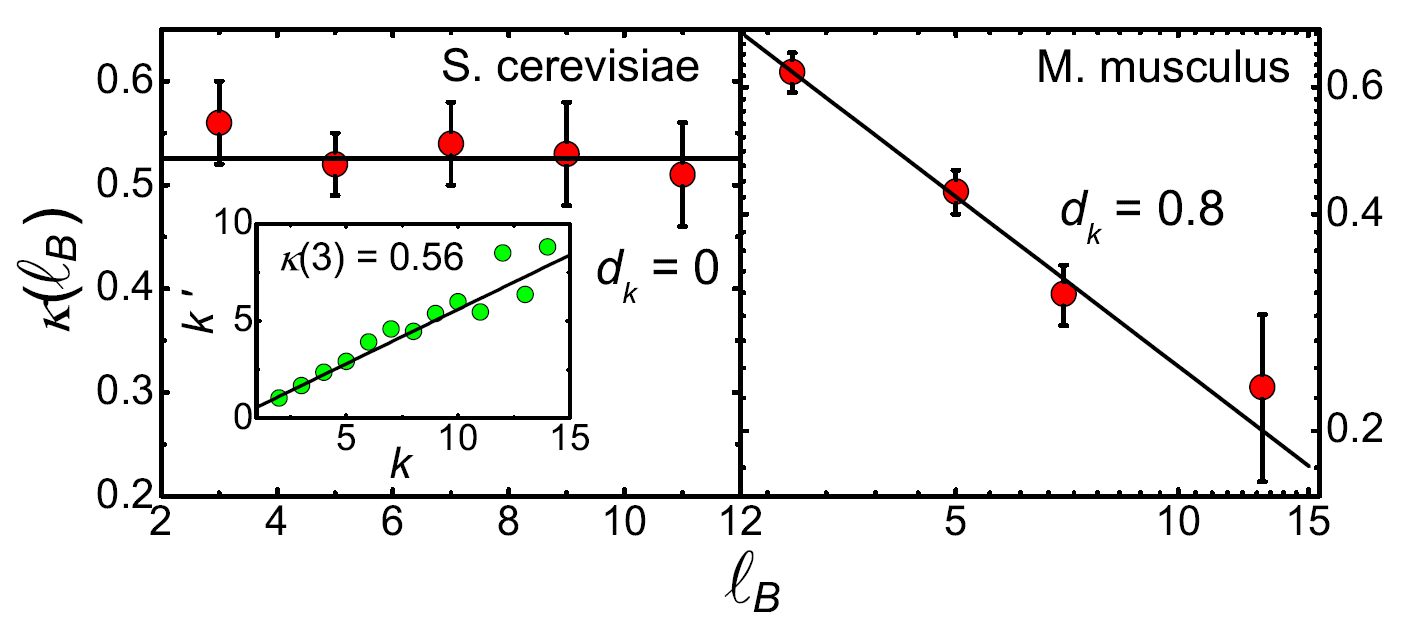

Supplement: Figure S10 — The scaling of vs. . The renormalized degree exponent is calculated according to Equation (4). As an example, the inset shows the renormalization relation for the case . (TIF) [file pone.0058134.s010.tif]

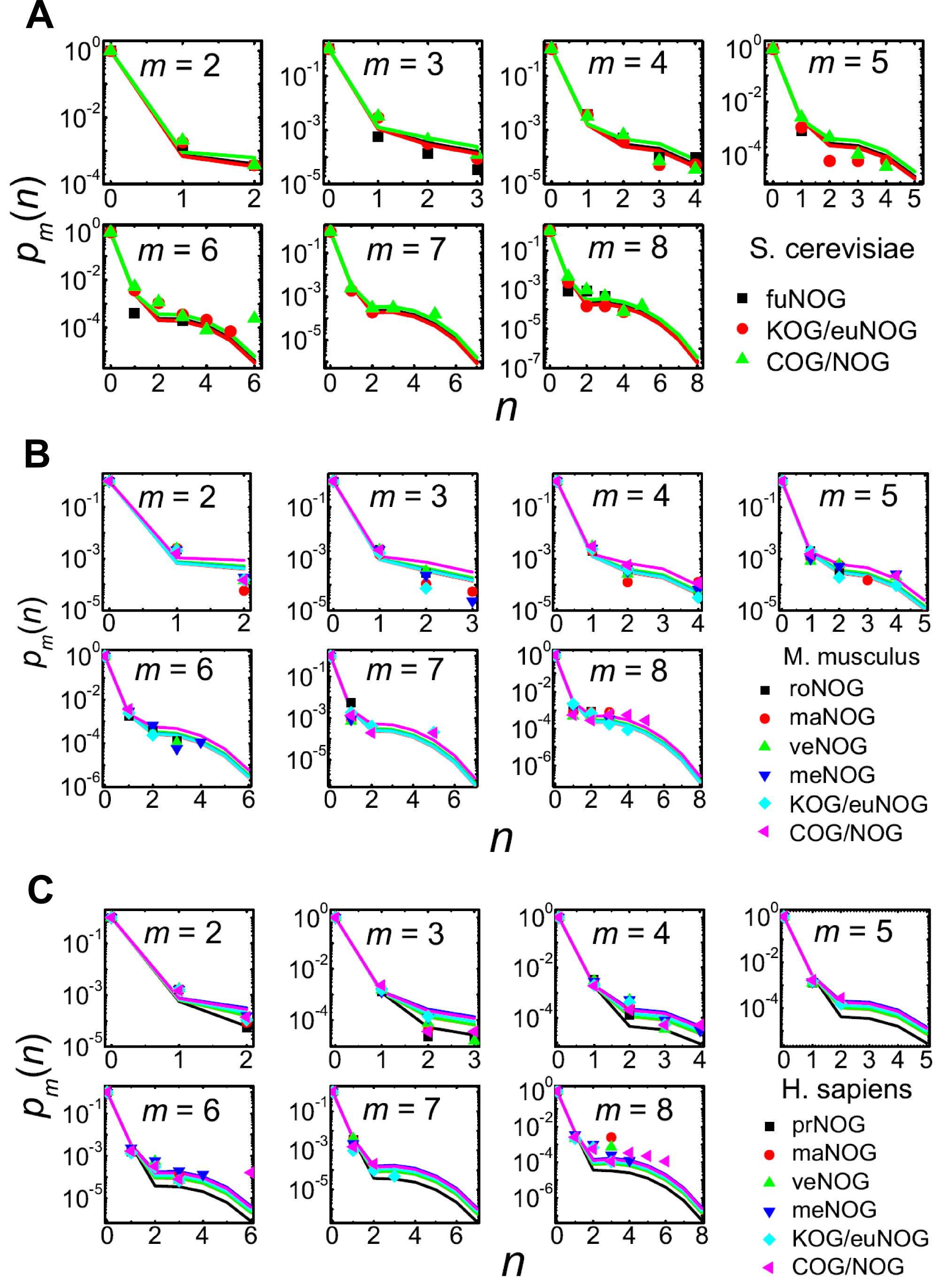

Supplement: Figure S11 — Fitting parameters and testing the duplication-divergence model. Fit of to the empirical data of (A) S. cerevisiae, (B) M. musculus, and (C) H. sapiens. The curves are the fitted theoretical values, and the scatters are the empirical data. The model and the data are in good agreement. Parameters , and (one for each time level ) of each species are determined from this fitting. (TIF) [file pone.0058134.s011.tif]
