# Supplementary material for: The Evolutionary Dynamics of Protein-Protein Interaction Networks Inferred from the Reconstruction of Ancient Networks
Source: PLoS One. 2013 Mar 20;8(3):e58134. doi: 10.1371/journal.pone.0058134 (PMC3603955; doi:10.1371/journal.pone.0058134)
Supplement: Table S1 — Node and interaction counts at each filter step. Numbers of proteins and interactions at each filter step preceding the network construction and analysis. Four different filters were applied: STRING experimental score , conservation on all evolutionary levels defined for the corresponding organism in eggNOG, filtering at the percolation threshold , and filtering at the percolation threshold and considering only the largest connected component. The largest component (which is also called giant component in the percolation literatures [62]) is required for the topological analysis. (PDF) [file pone.0058134.s012.pdf]

	Exp score > 0		Conserved on all eggNOG levels		After filtering at s_c*		After filtering at s_c* largest component	
Species	proteins	interactions	proteins	interactions	proteins	interactions	proteins	interactions
eco	2472	11016	2472	11016	873	2321	705	2209
sce	5388	124956	4197	75625	2144	6000	1609	5546
ath	1913	3513	1104	1792	727	905	404	618
cel	3370	6768	997	1391	485	438	249	271
dme	5376	8695	2213	2915	461	598	311	504
mmu	3513	5623	1321	1573	718	658	285	351
hsa	10617	51573	5445	19040	1891	2840	1365	2435
