# Supplementary material for: The Evolutionary Dynamics of Protein-Protein Interaction Networks Inferred from the Reconstruction of Ancient Networks
Source: PLoS One. 2013 Mar 20;8(3):e58134. doi: 10.1371/journal.pone.0058134 (PMC3603955; doi:10.1371/journal.pone.0058134)
Supplement: Table S2 — Divergence times. Estimated divergence times for the evolutionary levels in the eggNOG database. They represent the time point when the last common ancestor of a certain evolutionary level existed. Estimates are derived from the TimeTree database [74]. (PDF) [file pone.0058134.s013.pdf]

eggNOG level	Divergence time (million years)
COG/NOG	2313.2
KOG/euNOG	1369
fuNOG	798
meNOG	782.7
inNOG	366
veNOG	400.1
maNOG	92.4
roNOG	25.2
prNOG	6.4
